# The evolution of shame and guilt

**DOI:** 10.1371/journal.pone.0199448

**Published:** 2018-07-11

**Authors:** Libing Shen

**Affiliations:** 1 Institute of Neuroscience, Shanghai Institute for Biological Sciences, Chinese Academy of Sciences, Shanghai, P. R. China; 2 State Key Laboratory of Genetic Engineering, School of Life Sciences, Fudan University, Shanghai, P. R. China; 3 MOE Key Laboratory of Contemporary Anthropology, School of Life Sciences, Fudan University, Shanghai, P. R. China; 4 Interdisciplinary Research Center on Biology and Chemistry, Shanghai Institute of Organic Chemistry, Chinese Academy of Sciences, Shanghai, P. R. China; University of Waterloo, CANADA

## Abstract

Shame and guilt seem to be two synonymous moral emotions but actually lead to contrasting human behaviors or behavioral tendencies. Shame drives people to hide or deny their wrongdoings while guilt drives people to amend their mistakes. How shame and guilt evolved in humans is still obscure. Here we present a computer model featured with reciprocal altruism and gregarious lifestyle for studying this question. We tested ten different strategies in our model and the pairwise contests show that shame-driven-hiding strategy can dominate the other strategies such as tit-for-tat and Pavlov in more than half of parameter combinations. The mathematical analysis of our model demonstrates that shame-driven-hiding strategy is an evolutionary stable strategy within a group as long as hiding can let an individual evade the retaliations to his wrongdoings. However, the problem of hiding is that it reduces an individual’s social circle, i.e. living in a smaller group. Our analysis also shows that guilt-driven-amending strategy can outperform shame-driven-denying strategy at both individual and group levels if the cooperative behavior is sustainable within a group (*b*/(*b*-*c*) < *T*/*n*). Thus, we propose that shame is more adaptive at the individual level while guilt is more advantageous in the context of intergroup competition.

## Introduction

Moral emotions deeply influence our decision-making process and substantially regulate our social behaviors [1–3]. They differ from basic emotions such as happiness and sadness in terms of requiring the understanding of other peoples’ mentalities [4]. Therefore, they are also called social emotions. Shame and guilt are two particularly prominent moral emotions, because they serve as the conscience bases for different cultures [5].

Both shame and guilt are negative and painful emotions making them seem to be equivalents and thus their differences are constantly overlooked by many people [1, 2]. In Chinese traditional culture, people customarily used the concepts of shame and guilt interchangeably [6]. Shame and guilt do overlap in some psychological symptoms such as depression and self-derogation while they show different psychological trends, e.g. psychoticism vs. anxiety, and are accompanied by different physiological correlates, e.g. distinct immunological effects [7, 8]. Most importantly, shame and guilt correspond to contrasting human behaviors or behavioral tendencies [9–13].

To experience shame or guilt, a person must first understand that he has done something wrong or he has the intention to do something wrong, i.e. that he must have the knowledge of good and evil. If the mischief had been done, a shame-prone person would choose or attempt to hide or deny his wrongdoing while a guilt-prone person would choose or attempt to amend or compensate his wrongdoing [1]. So shame and guilt drive us to make different moral decisions which elicit different behavioral solutions for our misdeeds.

How shame and guilt evolved in human is still an open question. Shame is a more painful emotion than guilt and is associated with various maladaptive symptoms or abnormal behaviors such as eating disorders and self-injury [1, 7, 14, 15]. On the other hand, there is evidence showing that guilt is a more adaptive moral emotion in social life, which functions as a relationship enhancer [16, 17]. It is unclear why humans hold two seemingly similar but fundamentally different moral emotions at the same time and how guilt hasn’t replaced shame in human populations.

Here we present a computer model featuring reciprocal altruism and gregarious lifestyle for studying the evolution of shame and guilt in early human groups/societies. In our model, a virtual individual would randomly and repetitively engage in the cooperative behaviors (the iterated prisoner’s dilemma) with the other group members, from which he can benefit. The recurrent interactions between two group members also symbolizes gregariousness. The simulation results show that self-conscious strategies such as shame-driven-hiding and guilt-driven-amending generally outperform most non-self-conscious strategies in our model. Shame-driven-hiding can even dominate the other tested strategy in more than half of parameter combinations. Interestingly, our results also show that guilt-driven-amending usually grants a group with higher fitness payoff than shame-driven strategies at the same error rate. It indicates that guilt is more evolutionarily advantageous at group level. Our model explained why guilt and shame could coexist in human population. The scenario that an individual faces multiple opponents in an iterated prisoner’s dilemma (IPD) game catches the general characteristic of the social life within a community, especially for *Homo sapiens*.

## Methods

### The model

Human society is founded on cooperation [18]. Cooperation grants us reciprocal altruism --- we all gain benefits from our cooperative behaviors [19]. Modern human society relies on the sophisticated division of labor, which is an extensive form of cooperation. There were recurrent chances of social interactions between any two individuals within a group, especially in an early hunter-gather human community. These interactions could be generalized as the prisoner’s dilemma (cooperation or defection). In our model, the assumption of morality is that it is fundamentally wrong to defect in within-group cooperative behaviors.

**Fig 1:**
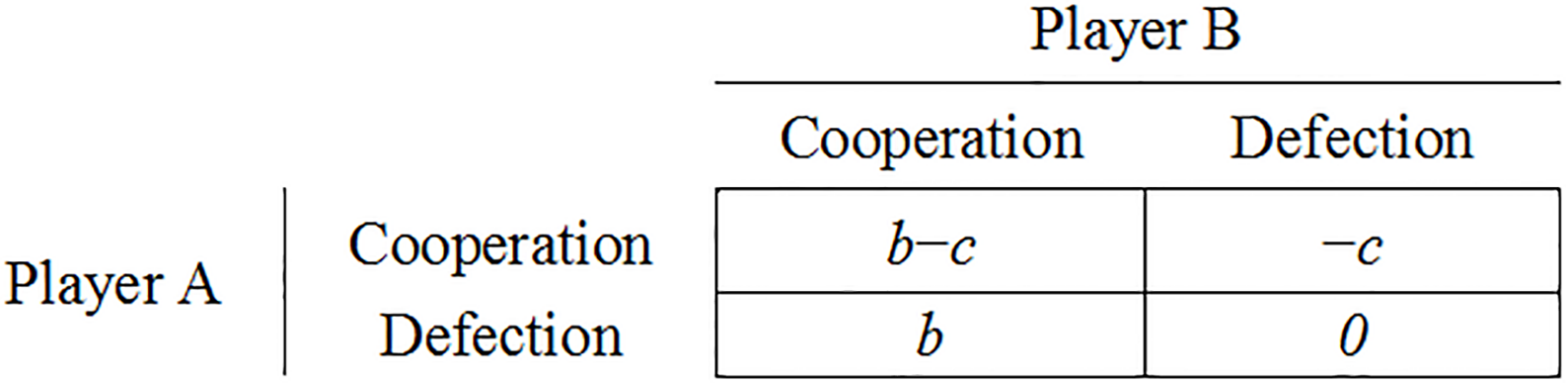
Payoff matrix of the “donation game”. The entries in the matrix refer to the payoffs of player A. The benefit of cooperation (*b*), the cost of cooperation (*c*), and *b* − *c* are greater than 0.

Assume a social group with *n* individuals. Any individual of the group repeatedly interacts with the other group members. The prisoner’s dilemma game is used to model cooperative and non-cooperative behaviors in these social interactions [19]. We use the payoff matrix of the “donation game” for modeling, which is a special case of the prisoner’s dilemma [20] (Fig 1). The benefit and cost in the donation game are interpreted as the gain and loss in an individual’s fitness. For modeling simplicity, we assume that there is no effect of kin selection on cooperation (a hunter-gather community was often made of genetically related individuals) and social interaction randomly occurs between any two individuals. Each individual has a unique name in order to be recognized by his opponent during the interaction. All group members have the same number of social interactions (*T*) and a fitness score of zero at the beginning of simulation.

### The tested strategies

We tested ten different strategies in our model. They are always-cooperate, always-defect, always-trembling, tit-for-tat, generous tit-for-tat, TFT-with-trembling-hand, shame-driven-hiding, shame-driven-denying, guilt-driven-amending, and Pavlov [19, 21, 22]. Table 1 shows the description for each strategy.

**Table 1:** Ten strategies tested in the model.

Strategy	Description
Always-cooperate	An always-cooperate individual always chooses to cooperate in social interaction, regardless of his opponent’s choice.
Always-defect	An always-defect individual always chooses to defect in social interaction, regardless of his opponent’s choice.
Always-trembling	An always-trembling individual randomly switches between cooperation and defection. His probability of cooperation or defection is 0.5 in each social interaction.
Tit-for-tat	A tit-for-tat individual always cooperates in the first round of social interaction with a new opponent and remembers his opponent’s choice. If he meets the opponent again, he will repeat his opponent’s choice in the previous round.
Generous tit-for-tat	A generous tit-for-tat individual basically uses the tit-for-tat strategy, but won’t retaliate on every defection. He has a certain probability ((*b*−*c*)/*c*) of cooperation when his opponent defects.
TFT-with-trembling-hand	A TFT-with-trembling-hand individual is basically a tit-for-tat individual except that he has a certain probability of random error (random defection) and doesn’t recall his error.
Shame-driven-hiding (self-conscious)	A shame-driven- hiding individual is basically a TFT-with-trembling-hand individual except that he remembers his error and tries to hide from it (avoid the interaction with the individual whom he defected on before).
Shame-driven-denying (self-conscious)	A shame-driven-denying individual is basically a TFT-with-trembling-hand individual except that he remembers his error and tries to deny it (deliberately defect on the individual whom he defected on before).
Guilt-driven-amending (self-conscious)	A guilt-driven-amending individual is basically a TFT-with-trembling-hand individual except that he remembers his error and tries to amend it (voluntarily cooperate with the individual whom he defected on before).
Pavlov*	A Pavlov individual uses a win-stay, lose-switch strategy. He only remembers his own choice. If he got *b*−*c* or *b* in social interaction, he would continue his choice. If he got–*c* or *0*, he would switch his choice.

* The individuals who adopt Pavlov strategy will also make random errors. They have a probability of randomly switching choice.

In our model, we introduce a probability of random error (*e*) to TFT-with-trembling-hand, shame-driven-hiding, shame-driven-denying, guilt-driven-amending, and Pavlov. For the TFT-with-trembling-hand, shame-driven-hiding, shame-driven-denying and guilt-driven-amending strategies, random error means switching to defection from cooperation. It simulates people’s wrongdoings in social life, e.g. lie, cheat, and steal, which are against morality and social norms. For the Pavlov strategy, random error means switching from current choice to the other one.

Shame-driven-hiding, shame-driven-denying, and guilt-driven-amending are the derived strategies of TFT-with-trembling-hand. They will retaliate upon those who defected on them in previous interactions while they also have a random probability of defection in each interaction. However, they differ from TFT-with-trembling-hand in being self-conscious. The individuals who adopt these strategies understand that they defected on someone while they should have cooperated with him and used different reactions to their errors. Thus, shame-driven-hiding, shame-driven-denying, and guilt-driven-amending are self-conscious strategies, whereas the remaining seven strategies are non-self-conscious ones.

The individuals who adopt shame-driven-hiding strategy use hiding to cope with their errors, i.e. avoiding the interaction with those whom they previously defected on. In our model, computer program will first randomly select a candidate of interaction for a shame-driven-hiding individual. If the candidate were defected on by him before, the shame-driven-hiding individual would skip this interaction and the computer program would randomly select another candidate for him from the rest group members. The individuals who adopt the shame-driven-denying strategy use denying to cope with their errors. In our computer simulations, we use defection to simulate the action of denying. Merriam-Webster’s dictionary defines one meaning of “deny” as refusing to grant [23]. In the donation game, if you want to refuse your opponent to receive the benefit from you, you will have to choose defection. In reality, denying symbolizes antagonistic behavior, which means defecting from truth and refusing to cooperate. In computer simulation, if a shame-driven-denying individual met an opponent whom he previously defected on, he would continue to choose defection in this interaction. The individuals who adopt the guilt-driven-amending strategy use amending to cope with their errors. In our model, we use voluntary cooperation to simulate the action of amending. If a guilt-driven-amending individual met an opponent whom he previously defected on, he would choose to cooperate with his opponent in this interaction. Even if his opponent defects on him in this interaction, he won’t retaliate in their next round of interaction because he understands that his opponent’s defection is due to his previous error. For a guilt-driven-amending individual, his voluntary cooperation simulates the redemption in reality.

Thus, in our model, we use interaction avoidance, deliberate defection, and voluntary cooperation to simulate the behavior of hiding, denying, and amending, respectively.

However, there are two problems caused by the introduction of the probability of random error in our model.

First, for a shame-driven hiding individual, if the probability of random error is very high and/or the group size is very small, sooner or later he will defect on everyone in the group and exhaust the potential for hiding. Our solution is that if a shame-driven hiding individual exhausts the potential candidate for hiding, he will switch from hiding to denying (defection) in his rest interactions. Thus, the shame-driven-hiding strategy is actually the same as the shame-driven-denying strategy in such a situation. For shame-driven individuals, when hiding is not an option, denying is the only choice. The reason why we set the shame-driven hiding strategy like this is that people rarely change their mentality in their life.

Second, because errors are randomly generated for tit-for-tat based strategies in computer simulation, it is inevitable that two individuals adopting these strategies would both choose to defect in the same interaction. It creates a problem in our simulation when a player should retaliate. In our computer simulations, if two individuals mutually defected on each other in the same interaction, they wouldn’t choose to retaliate in their next round of interaction. Humans have a psychological tendency called loss aversion [24]. It shows that people would rather avoid losses than make gains. The psychological tendency of loss aversion phenomenon could be also seen in the other primates [25]. In the donation game, if both parties decide to defect, they both get nothing but have no loss. Since people hate loss more than no gain, retaliation is based on loss in our model. Thus, an individual who adopts the tit-for-tat based strategies only retaliates (deliberately chooses to defect) when he chooses to cooperate while his opponent chooses to defect in their previous interaction.

### Pairwise contests and tested parameters

We performed pairwise contests among ten tested strategies in our model. The contest was set in a group scenario (one vs. many) and we gradually increased the individuals of one strategy and decreased the ones of the other in the group. Each contest with different ratios of the individuals from two strategies was repeated 100 times in order to get the average fitness payoffs for two competing strategies.

The number of social interactions (*T*) for each group member was set to 200 as in the original IPD tournament [19]. Three different sets of benefit (*b*) and cost (*c*), four different group sizes (*n*) and four different probabilities of random error (*e*) were tested in our model. They are listed as follows: *b* = 1 and *c* = 0.75, *b* = 1 and *c* = 0.5, *b* = 1 and *c* = 0.25, *n* = 10, 20, 50 and 100, *e* = 0.01, 0.05, 0.1 and 0.2.

## Results

**Fig 2:**
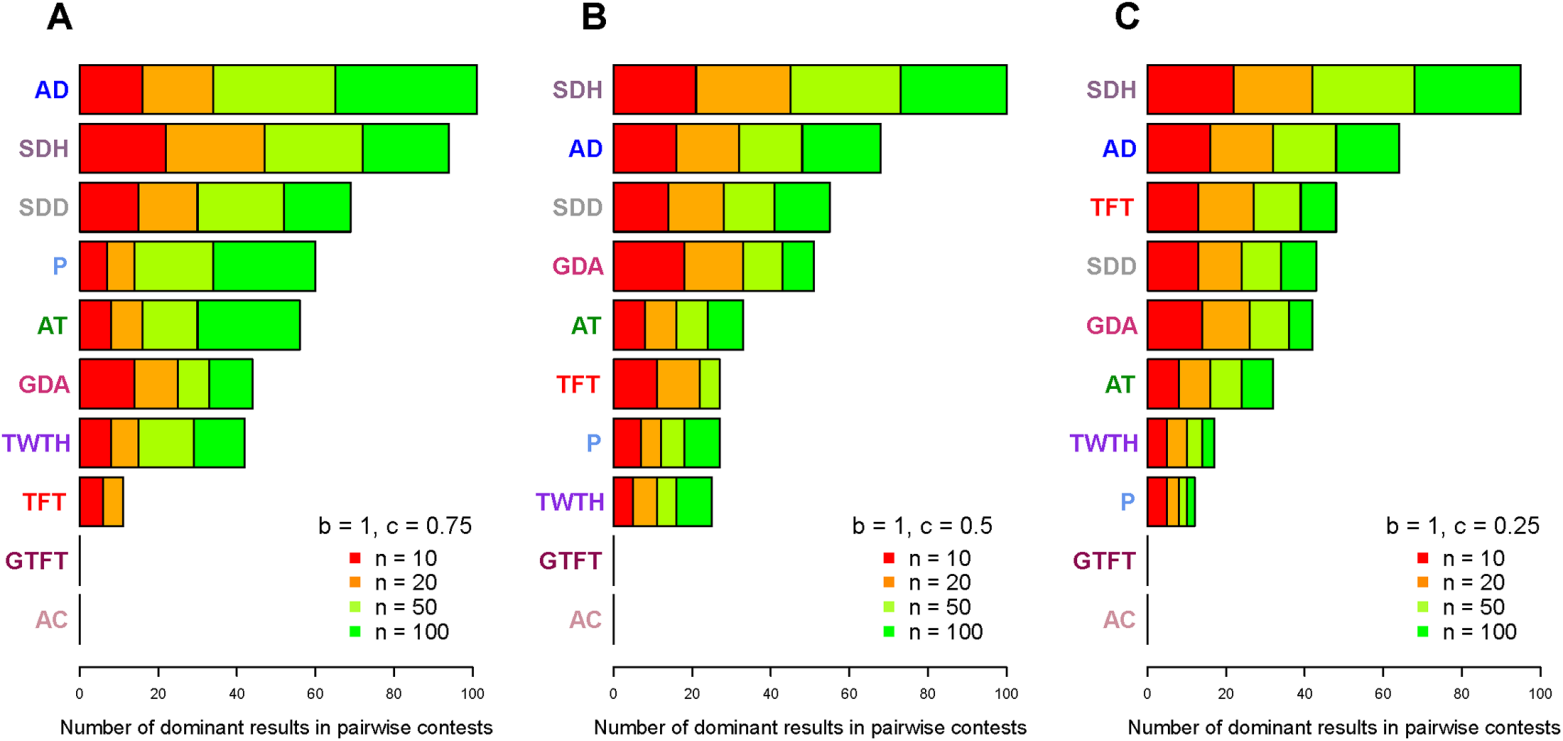
The number of dominant results for nine strategies tested in pairwise contests. AC stands for always-cooperate. AD stands for always-defect. AT stands for always-trembling. TFT stands for tit-for-tat. GTFT stands for generous tit-for-tat. TWTH stands for TFT-with-trembling-hand. SDH stands for shame-driven-hiding. SDD stands for shame-driven-denying. GDA stands for guilt-driven-amending. P stands for Pavlov. (A) When *b* = 1 and *c* = 0.75, the ranking of nine strategies. (B) When *b* = 1 and *c* = 0.5, the ranking of the strategies. (C) When *b* = 1 and *c* = 0.25, the ranking of nine strategies.

We tested ten strategies and 48 different parameter combinations in our model, so there were total 432 pairwise-contest results for each strategy (please see the appendix tables in S1 File for the evolution of shame and guilt). Under the hypothesis that natural selection favors the strategy with a higher fitness payoff, these results can be classified into seven different possible selection dynamics (see S1 File for the evolution of shame and guilt). The most relevant dynamic for this study is that one strategy dominates another. If A strategy dominates B strategy, the individuals who adopt A strategy will always have a higher fitness payoff than the individuals who adopt B strategy and thus A individuals are more likely to survive and reproduce than B individuals within one group. We counted the number of dominant results for each strategy and classified these results according to different benefit and cost ratios (Fig 2).

When *b*/*c* = 4/3 (*b* = 1, *c* = 0.75; Fig 2A), the strategy with the most dominant results is always-defect; the second one is shame-driven-hiding and the third one is shame-driven-denying. At this benefit-cost ratio, we found that always-defect dominates the other strategies when group size is equal to or larger than 50 (*n* = 50 and 100), where *b*/(*b-c*) ≥ *T*/*n*. As long as group size was smaller than 50 (*b*/(*b*-*c*) < *T*/*n*) and hiding is a viable option (*Te* < *n*−1, an individual’s errors don’t exceed group size), shame-driven-hiding is the evolutionary stable strategy against the other tested strategies within a group (please see the appendix tables in S1 File for the evolution of shame and guilt).

When *b*/*c* = 2 (*b* = 1, *c* = 0.5; Fig 2B), the strategy with the most dominant results is shame-driven-hiding and the second one is always-defect; shame-driven-deny and guilt-driven-amending hold the third and the fourth position. At this benefit-cost ratio, a shame-driven-hiding population is only vulnerable to the invasion of always-defect when group size is equal to 100 and error rate is equal to 0.2; except that, as long as hiding is a viable option (*Te* < *n*−1), shame-driven-hiding is the evolutionary stable strategy against the other tested strategies (please see the appendix tables in S1 File for the evolution of shame and guilt).

When *b*/*c* = 4 (*b* = 1, *c* = 0.25; Fig 2C), the strategy with the most dominant results is shame-driven-hiding; the second one is always-defect and the third one is tit-for-tat. At this benefit-cost ratio, shame-driven-denying and guilt-driven-amending hold the fourth and fifth position. Shame-driven-denying surpasses guilt-driven-amending when *n* = 100, while guilt-driven-amending outperforms shame-driven-denying when *n* = 10, 20, and 50. In pairwise contests, always-defect can dominate the other tested strategies as long as the ratio of defection payoff to cooperation payoff is not smaller than the number of average interactions between any two group members (*b*/(*b-c*) ≥ *T*/*n*); when *b*/(*b-c*) < *T*/*n*, shame-driven-hiding is the evolutionary stable strategy against the other tested strategies if hiding is a viable option (*Te* < *n* −1).

In pairwise contests, we can see that self-conscious strategies usually outperform most non-self-conscious strategies including TFT-with-trembling-hand and Pavlov, especially when the benefit-cost ratio is large. We also ran simulations for multiple strategies competing in a group at the same time (Table 2). We found that tit-for-tat, shame-driven-hiding and guilt-driven-amending are usually the strategies with the highest fitness payoff.

**Table 2:** The average fitness payoff for ten strategies competing in a group under the conditions that group size is 50 (*n* = 50), benefit equals 1 and cost equals 0.25 (*b* = 1 and *c* = 0.25).

Strategy	Average fitness payoff					
n = 50	e = 0.01	e = 0.05	e = 0.1	e = 0.2	e = 0.3	e = 0.4
AC (n_AC_ = 5)	117.404	110.788	103.612	90.068	81.006	72.498
AD (n_AD_ = 5)	83.232	83.312	83.17	82.274	81.618	81.268
AT (n_AT_ = 5)	97.628	95.851	93.96	89.362	84.803	80.479
TFT (n_TFT_ = 5)	123.608	117.078	110.562	99.483	91.044	85.198
GTFT (n_GTFT_ = 5)	118.972	112.194	105.212	92.772	82.834	75.121
TWTH (n_TWTH_ = 5)	122.524	114.576	106.254	95.685	89.291	83.529
SDH (n_SDH_ = 5)	123.592	117.873	111.192	98.136	86.547	76.087
SDD (n_SDD_ = 5)	122.459	113.679	105.273	94.249	87.894	83.104
GDA (n_GDA_ = 5)	122.813	115.017	107.288	97.381	90.564	85.285
P (n_P_ = 5)	97.999	96.521	93.662	89.420	84.771	80.705

Note: Every strategy has five individual in this group, e.g. n_AC_ = 5. The average fitness payoff in this table is based on 100 simulations. AC stands for always-cooperate. AD stands for always-defect. AT stands for always-trembling. TFT stands for tit-for-tat. GTFT stands for generous tit-for-tat. TWTH stands for TFT-with-trembling-hand. SDH stands for shame-driven-hiding. SDD stands for shame-driven-denying. GDA stands for guilt-driven-amending. P stands for Pavlov.

Our mathematical analyses of pairwise contests show that shame-driven-hiding is the evolutionary stable strategy against all the other tested strategies as long as the hiding behavior can work. A previous study showed that tit-for-tat is the long-lasting champion of the IPD Competition [19]. Assume that player A is a tit-for-tat player who doesn’t make any error and player B is his opponent who uses a random strategy with an error rate of *e*_*b*_. Because A only uses defections as retaliations to his opponent’s defections, A’s payoff with opponent B in the donation game is *E*(*A*,*B*) = *T*[(*1*−*e*_*b*_)*b*−(1−*e*_*a*_)*c*] (here *e*_*a*_ = *e*_*b*_ and *T* = ∞). If player B uses shame-driven-hiding strategy and hiding can always keep B from being retaliated against by his opponent (certainly, hiding won’t work if there are only two players), B’s payoff with player A in the donation game is *E*(*B*,*A*) = *T*[*b*−(1−*e*_*b*_)*c*] and A’s payoff becomes *E*(*A*,*B*) = *T*[(1−*e*_*b*_)*b*−*c*]. Here *E*(*B*,*A*) > *E*(*A*,*B*), hence shame-driven-hiding can dominate tit-for-tat as long as hiding can let an individual escape from retaliation. Our mathematical analyses also show that under the same error rate guilt-driven-amending strategy can dominate shame-driven-denying strategy in a group as long as *T* is large enough (please see the detailed mathematical analyses in S1 File for the evolution of shame and guilt).

**Fig 3:**
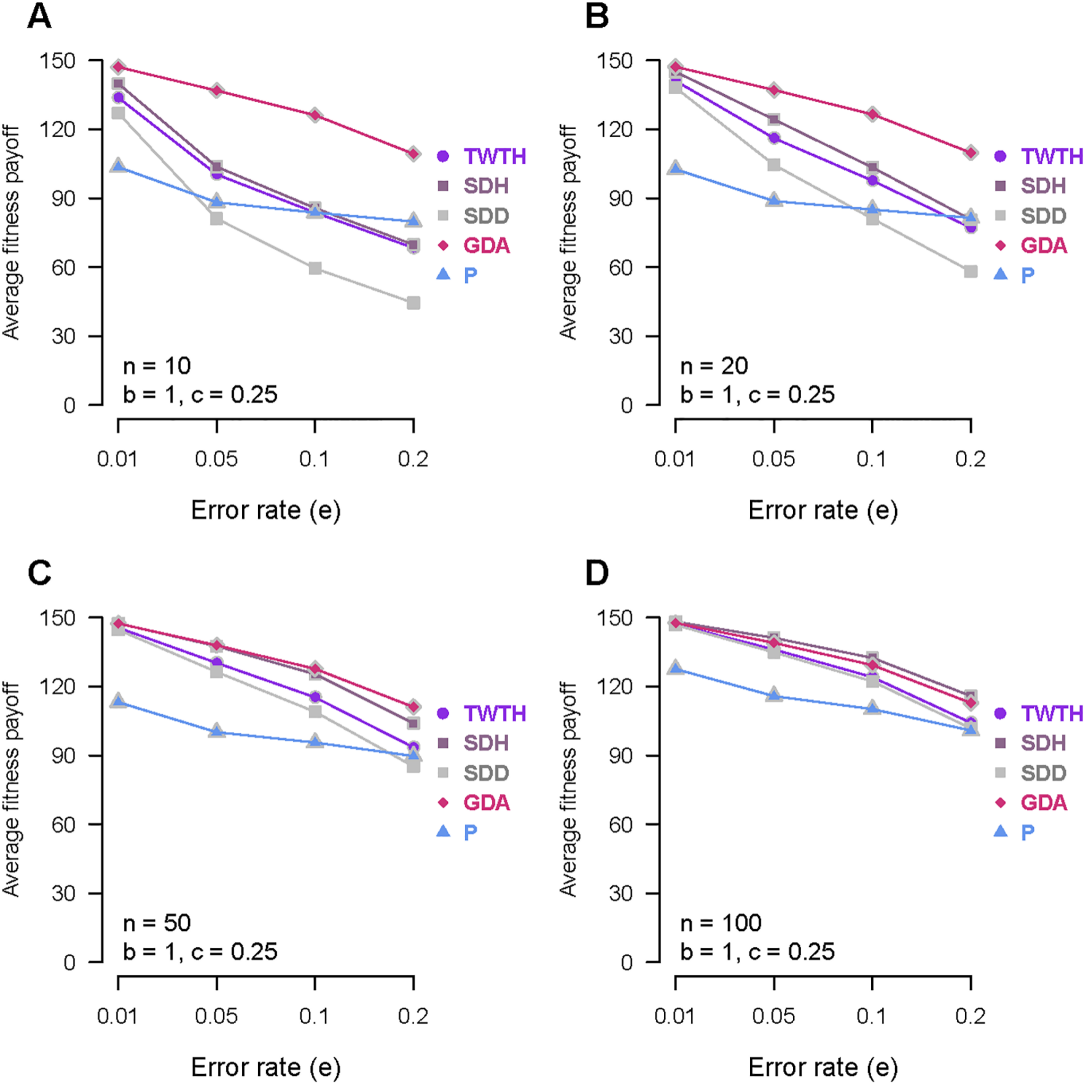
The average fitness payoff for five homogeneous groups which adopt the error-prone strategies at four different error rates. TWTH stands for TFT-with-trembling-hand. SDH stands for shame-driven-hiding. SDD stands for shame-driven-denying. GDA stands for guilt-driven-amending. P stands for Pavlov. (A) When *b* = 1, *c* = 0.25 and *n* = 10. (B) When *b* = 1, *c* = 0.25 and *n* = 20. (C) When *b* = 1, *c* = 0.25 and *n* = 50. (D) When *b* = 1, *c* = 0.25 and *n* = 100.

Human beings not only compete at the individual level but also at the group level [26]. Thus, we compared the group’s average fitness payoffs of five error-prone strategies. In group-based comparisons, all members of the group adopt the same strategy. The simulation results are shown in Fig 3. When group size is small (*n* = 10 or *n* = 20), guilt-driven-amending group has the highest average fitness payoff at any error rate and shame-driven-hiding group has higher average fitness payoff than shame-driven-denying group (Fig 3A and 3B). When group size is large (*n* = 50 or *n* = 100), guilt-driven-amending group and shame-driven-hiding group have similar average fitness payoff; guilt-driven-amending group has relatively better performance than shame-driven-hiding group when *n* = 50, while shame-driven-hiding group has relatively better performance than guilt-driven-amending group when *n* = 100 (Fig 3C and 3D). Among three self-conscious strategies, the group which adopts shame-driven-denying always has the worst average fitness payoff and is even worse than TFT-with-trembling-hand group whose members are non-self-conscious.

## Discussion

The result that the shame-driven-hiding group has a similar or slightly higher average fitness payoff than the guilt-driven-amending group seems to propose that the behavior of hiding is not only beneficial at the individual level but also at the group level when group size is large (*n* = 100). However, in our model, hiding is achieved through artificially forbidding the interaction between a shame-driven-hiding individual and his victims. By doing so, we violated the randomness assumption of social interactions for shame-driven-hiding strategy while the other tested strategies didn’t enjoy such a privilege. This privilege gives shame-driven-hiding strategy a fitness advantage in simulations. Nevertheless, hiding is not always a viable option in real life as it was in a deterministic computer program. When confronted with the retaliation for his early transgression in real life scenarios, a shame-prone person would naturally choose to deny his wrongdoings if his hiding behavior failed. Our results have already shown that denying as an antagonistic behavior is inferior to amending in terms of individual and group average fitness payoff. Moreover, even if hiding can let a person escape from his punishment, it will reduce his number of social connections. Living in a smaller social group is surely disadvantageous in intergroup competition as the strength is in numbers. Thus, our simulation results propose that guilt is more advantageous than shame in the context of intergroup competition.

In our simulation, the group made of the entirely error-free (always-cooperate or tit-for-tat) individuals has the highest fitness payoff at both individual and group levels. The problem is that to err is a part of human nature. Hiding, denying and amending are the different behaviors that we use to cope with our errors. Especially, hiding and denying are two different behaviors stemming from the same cognition. Our simulation results propose that hiding is a more adaptive behavior than denying. It explains why shame is tightly associated with low self-esteem which causes social withdrawal [1, 27]. Shame also has a peculiar link with anger which leads to costly antagonistic behaviors such as denying [9, 28, 29]. We infer that a shame-prone person would only choose to deny his errors when he is unable to hide from the shame-inducing encounters, although he might experience low self-esteem and anger at the same time.

In addition, our simulations show that the tit-for-tat-based strategies excelled Pavlov in most parameter combinations and the generous tit-for-tat is unable to dominate any strategy in all pairwise contests. This result is consistent with the previous study that tit-for-tat is one of our psychological machineries and explains why unconditional forgiveness is rare in social life [30]. Since most human beings act in a tit-for-tat fashion and unconditional forgiveness is evolutionarily unstable, self-conscious emotions such as shame and guilt could evolve as psychological heuristic mechanisms to tackle our ever-present errors in a complicated social environment which have many opponents instead of one. Moreover, our simulation results show that there is no single strategy that can dominate the other strategies under all parameter combinations in our pairwise contests. Real life is much more complicated than our simple model. Thus, we conclude that in a human society individual mentalities must be diversified, i.e. human’s psychology exhibits a mixed strategy equilibrium at the group level.

There is evidence that genes play a role in empathy [31]. If genetic predisposition also played a role in shaping moral emotions which serve as the substratum for cultures, it is hypothetical that the genetic variation among different human ethnic groups could partly account for their cultural differences and intergroup competition would have further solidified these differences and catalyzed the gene-culture co-evolution. However, this hypothesis is difficult to directly test on scientific grounds. The intergroup competition among human societies is an ongoing multilevel competition, e.g. at scientific and technical level, and thus it is also unfathomable through simple computer simulations. Furthermore, to excel in intergroup competition, every human society has a unique culture system to regulate its member’s behaviors in order to reduce its member’s error rate (the actions deemed against social norms). Naturally, a shame based system is more inclined to rely on inner pain and role models to curb its member’s instinct to defect while a guilt based one is more likely to lean on conscience and a code of conduct. Although our analyses propose that guilt is more advantageous than shame in intergroup competition, a shame-based culture system could mitigate the detrimental effect of shame in social life and intergroup competition if it sufficiently controlled its member’s behavior.

## Supporting information

S1 File The supporting information for the evolution of shame and guilt. (DOCX)
