# Supplementary material for: The evolution of shame and guilt
Source: PLoS One. 2018 Jul 11;13(7):e0199448. doi: 10.1371/journal.pone.0199448 (PMC6040729; doi:10.1371/journal.pone.0199448)
Supplement: S1 File — (DOCX) [file pone.0199448.s001.docx]

Supporting information for

**The evolution of shame and guilt**

Libing Shen^1,2,3*^

Author Affiliations

^1^Institute of Neuroscience, Shanghai Institute for Biological Sciences, Chinese Academy of Sciences, Shanghai, 200031, P.R. China

^2^State Key Laboratory of Genetic Engineering and MOE Key Laboratory of Contemporary Anthropology, School of Life Sciences, Fudan University, Shanghai, 200433, PR China

^3^Interdisciplinary Research Center on Biology and Chemistry, Shanghai Institute of Organic Chemistry, Chinese Academy of Sciences, Shanghai, 200032, P. R. China

^*^Corresponding author: Libing Shen, email: [libingshen12@fudan.edu.cn](mailto:libingshen12@fudan.edu.cn)

Contents:

1. *Detailed description of pairwise contest and tested parameters*
2. *Selection dynamics in pairwise contests*
3. *The results of pairwise contests*
4. *Mathematical analysis of pairwise contests*
5. *Multiple strategies competing in a group at the same time*
6. *The group’s average fitness payoffs of five strategies under different benefit to cost ratios*
7. *Source code and contest results*
8. *References*
9. *Appendix: pairwise-contest results organized in table format*
10. *Detailed description of pairwise contest and tested parameters*

In order to assess their performance in donation game, we ran pairwise contests for ten strategies in our computer simulations. The pairwise contest is held as follows:

1. Two different strategies compete in a fixed-sized group;
2. We gradually (one-by-one) increase the individuals of one strategy from zero to group size and decrease the individuals of another in the same group;
3. Because errors are randomly generated in simulation, each contest with different ratios of the individuals from two strategies are repeated 100 times in order to get the average fitness payoff for each strategy.

The parameters we tested in pairwise contests are as follows:

1. Each virtual individual with a beginning fitness of zero at the start of each contest;
2. Each virtual individual has 200 rounds of social interaction (*T* = 200, rounds of donation game), which is the same as Axelrod and Hamilton’s computer tournament of iterated prisoner’s dilemma [[1](#_ENREF_1)];
3. We use three different benefit and cost combinations in contest. They are *b* = 1 and *c* = 0.75, *b* = 1 and *c* = 0.5, and *b* = 1 and *c* = 0.25;
4. We set four different group sizes for contest. They are *n* = 10, *n* = 20, *n* = 50, and *n* = 100;
5. We also set four different error rates. They are *e* = 0.01, *e* = 0.05, *e* = 0.1, and *e* = 0.2. For an individual who adopts TFT-with-trembling-hand, shame-driven-hiding, shame-driven-denying, guilt-driven-amending, and Pavlov strategies, he will have the number of random errors of *T*×*e* in social interactions. For example, a TFT with trembling hand individual with 0.05 error rate will have 10 random errors in contest (200×0.05 = 10).

We tested ten different strategies in our model. There are total 45 pairwise contests among ten strategies which produced 90 results. We also tested three benefit and cost combinations, four group sizes, four error rates, so the final number of results is 4320 (90×3×4×4).

1. *Selection dynamics in pairwise contests*

There are total 4320 results for ten strategies in pairwise contest and each strategy has 432 results. Under the hypothesis that natural selection favors the strategy with a higher fitness payoff, these results can be classified into seven different selection dynamics. Five of them have already been discussed in Nowak’s book [[2](#_ENREF_2)].

In Figure A, we can see that in a group with 20 individuals, the always-defect individuals always have a higher fitness payoff than the always-cooperate ones no matter how many group members adopt always-cooperate strategy. The result shows that always-defect strategy dominates always-cooperate strategy.


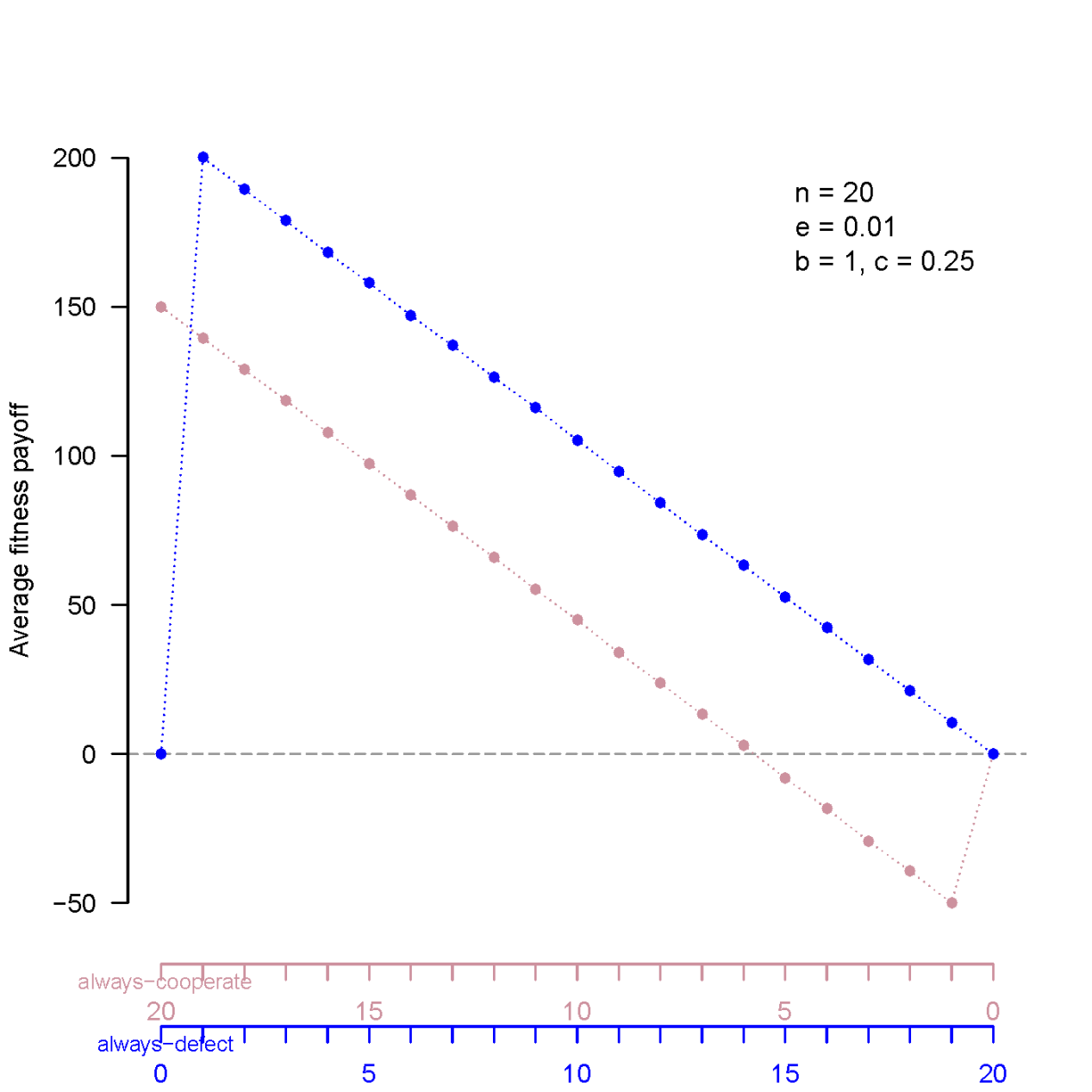


**Figure A. Always-cooperate against always-defect under the conditions that group size equals 20 (*n* = 20), benefit equals 1 and cost equals 0.25 (*b* = 1 and *c* = 0.25). Two *x* axes show that the number of always-cooperate individuals is decreasing from 20 to 0 and the number of always-defect individuals is increasing from 0 to 20. The individuals from two strategies always sum up to 20. *Y* axis show the average fitness payoff for always-cooperate and always-defect. Please note that error rate (*e* = 0.01) doesn’t apply to always-cooperate and always-defect.**

Dynamic 1: A strategy dominates B strategy. A can invade a B group and takes over the group. A is an evolutionarily stable strategy (ESS).

Dynamic 2: B strategy is dominated by A strategy. A can eliminate B in a group and B is not an ESS.


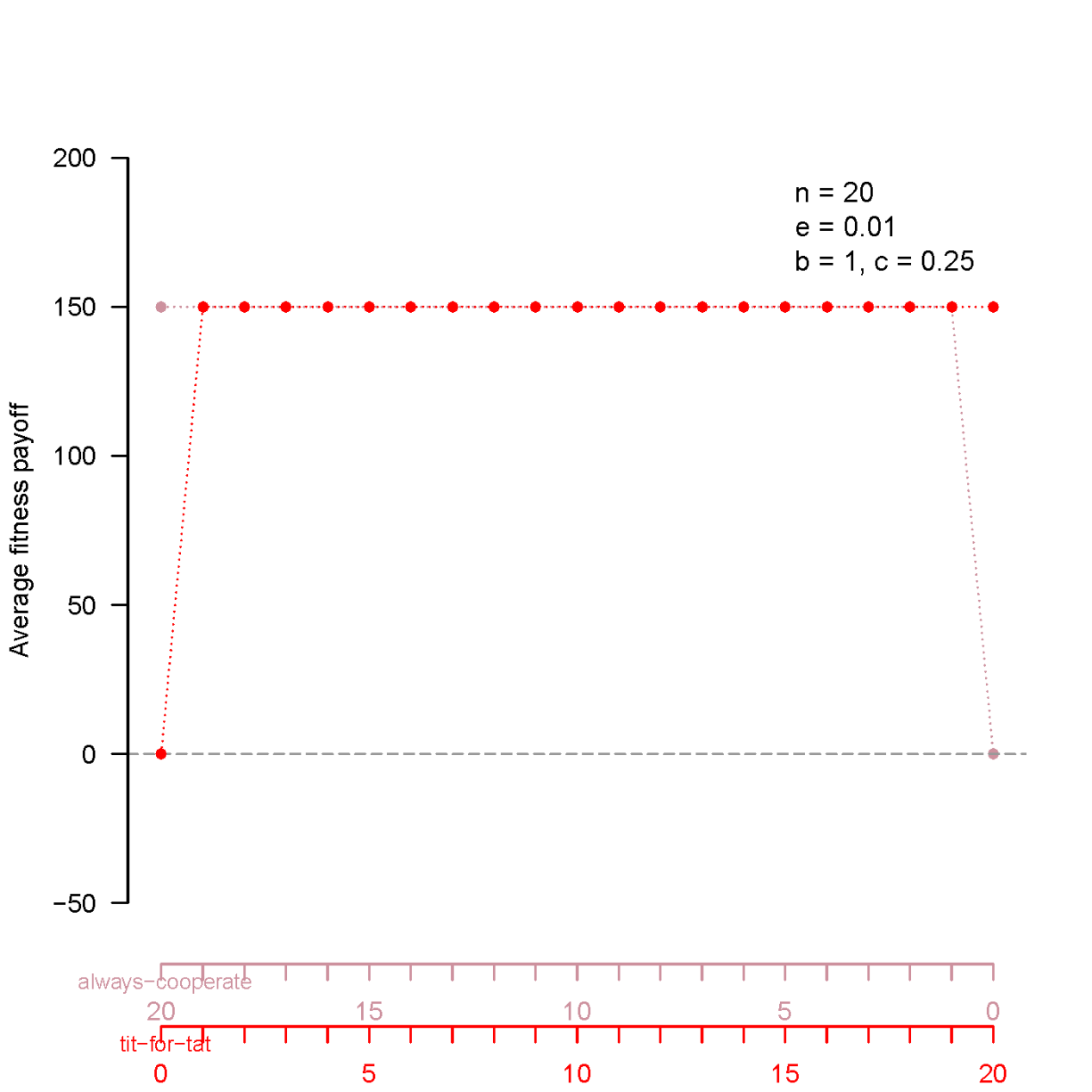


**Figure B. Always-cooperate against tit-for-tat under the conditions that group size equals 20 (*n* = 20), benefit equals 1 and cost equals 0.25 (*b* = 1 and *c* = 0.25). Two *x* axes show that the number of always-cooperate individuals is decreasing from 20 to 0 and the number of tit-for-tat individuals is increasing from 0 to 20. The individuals from two strategies always sum up to 20. *Y* axis show the average fitness payoff for always-cooperate and tit-for-tat. Please note that error rate (*e* = 0.01) doesn’t apply to always-cooperate and tit-for-tat.**

In Figure B, we can see that no matter what kind of ratio always-cooperate and tit-for-tat individuals are within a group, they have exactly the same pay-off.

Dynamic 3: A and B strategies are neutral. A and B have the same payoff and neither of them is a pure ESS in a group. The group is in the status of mixed-strategy equilibrium (mixed ESS). There is no equilibrium point within the group (A and B individuals can be at any ratio).


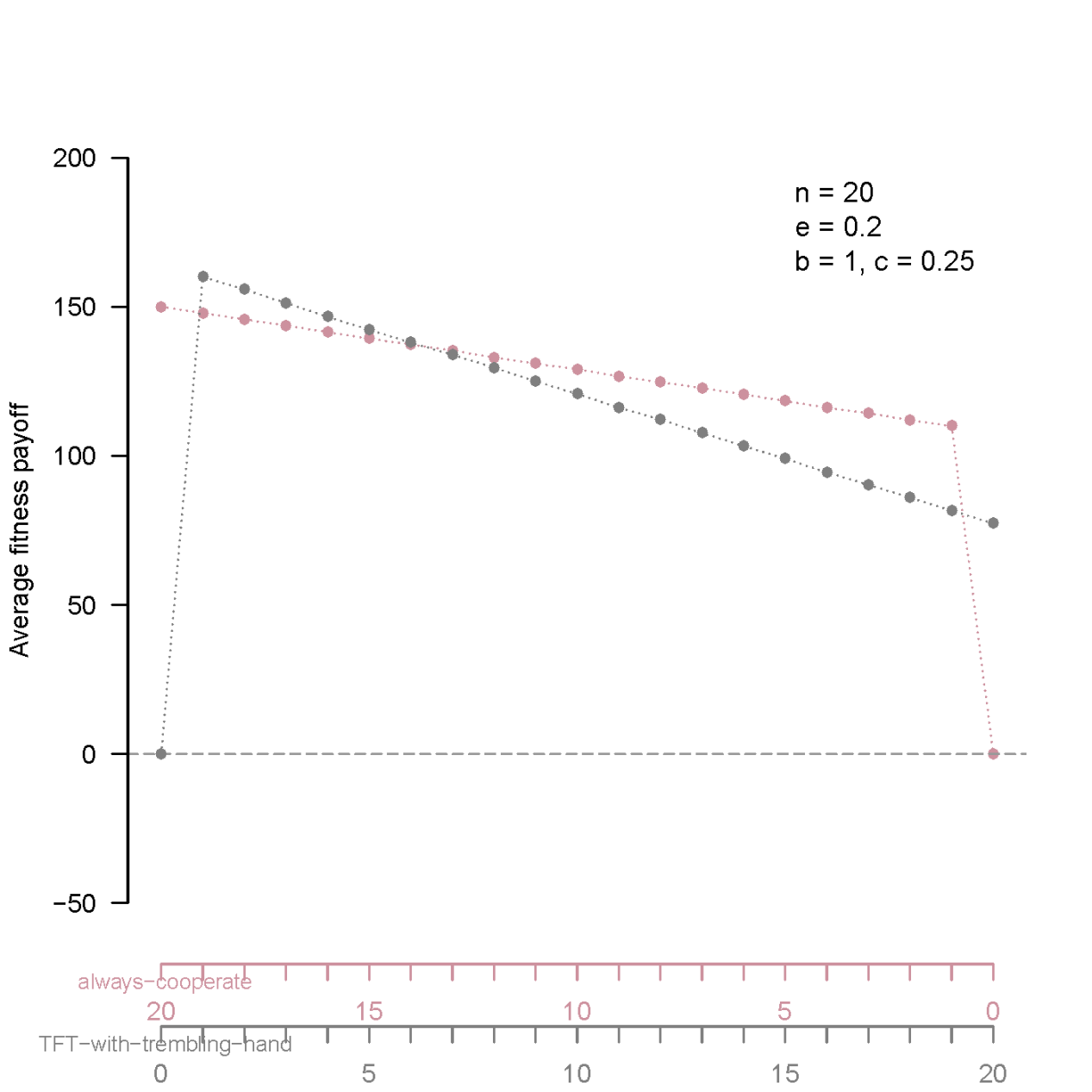


**Figure C. Always-cooperate against TFT-with-trembling-hand under the conditions that group size equals 20 (*n* = 20), benefit equals 1 and cost equals 0.25 (*b* = 1 and *c* = 0.25). Two *x* axes show that the number of always-cooperate individuals is decreasing from 20 to 0 and the number of TFT-with-trembling-hand individuals is increasing from 0 to 20. The individuals from two strategies always sum up to 20. *Y* axis show the average fitness payoff for always-cooperate and TFT-with-trembling-hand. Please note that error rate (*e* = 0.2) only applies to TFT-with-trembling-hand.**

In Figure C, we can see that in a group of 20 members when the number of TFT-with-trembling-hand individuals is less than 6, their average fitness payoff is higher than that of always-cooperate individuals. However, when the number of TFT-with-trembling-hand individuals is more than 6, their average fitness payoff starts to be lower than that of always-cooperate individuals. The group is at a fitness payoff equilibrium point when it has 6 TFT-with-trembling-hand and 14 always-cooperate individuals.

Dynamic 4: A and B strategies co-exist. When A can invade a B group and vice versa, the invading strategy initially has a higher payoff than the native one while the native strategy can resist the progress of invading strategy and lower its pay-off. Neither A nor B is a pure ESS. Finally, the group made of A and B will reach a stable equilibrium, which is a mixed ESS.


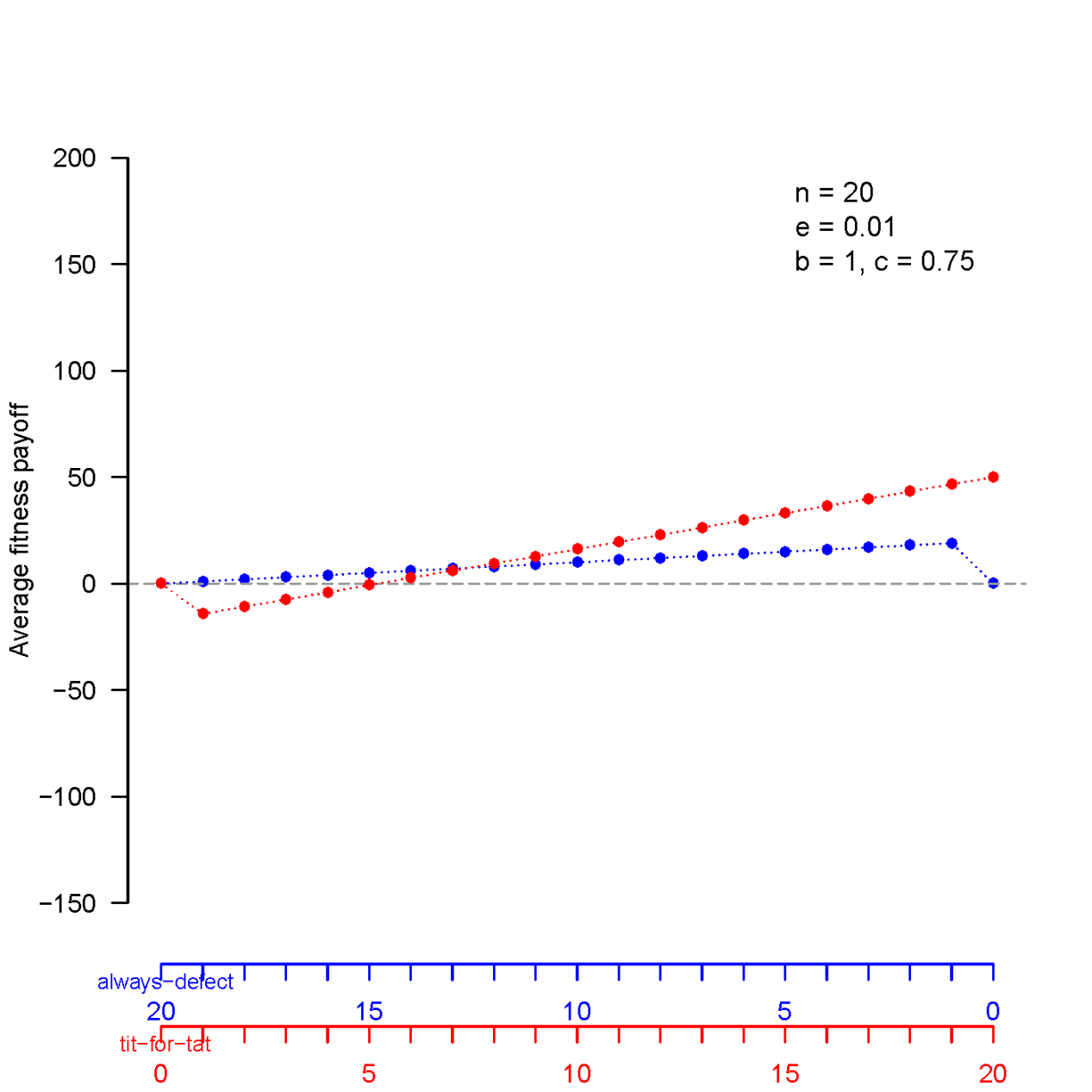


**Figure D. Always-defect against tit-for-tat under the conditions that group size equals 20 (*n* = 20), benefit equals 1 and cost equals 0.75 (*b* = 1 and *c* = 0.75). Two *x* axes show that the number of always-defect individuals is decreasing from 20 to 0 and the number of tit-for-tat individuals is increasing from 0 to 20. The individuals from two strategies always sum up to 20. *Y* axis show the average fitness payoff for always-defect and tit-for-tat. Please note that error rate (*e* = 0.01) doesn’t apply to always-defect and tit-for-tat.**

In Figure D, we can see that in a group of 20 members when the number of always-defect individuals is more than 13, their average fitness payoff is higher than that of tit-for-tat individuals. However, when the number of always-defect individuals is less than 13, their average fitness payoff starts to be lower than that of tit-for-tat individuals. There is an unstable equilibrium point in the group when the number of always-defect individuals is 13 and the number of tit-for-tat individuals is 7.

Dynamic 5: A and B strategies are bi-stable. When A cannot invade a B group and vice versa, they are in a bi-stable status. As which strategy will be ESS and take over the whole group depends on its initial frequency in the group. There exists an unstable equilibrium point in the group made of A and B. At this point, the group is a mixed ESS and will eventually converge to a pure ESS (A or B) if disturbed.


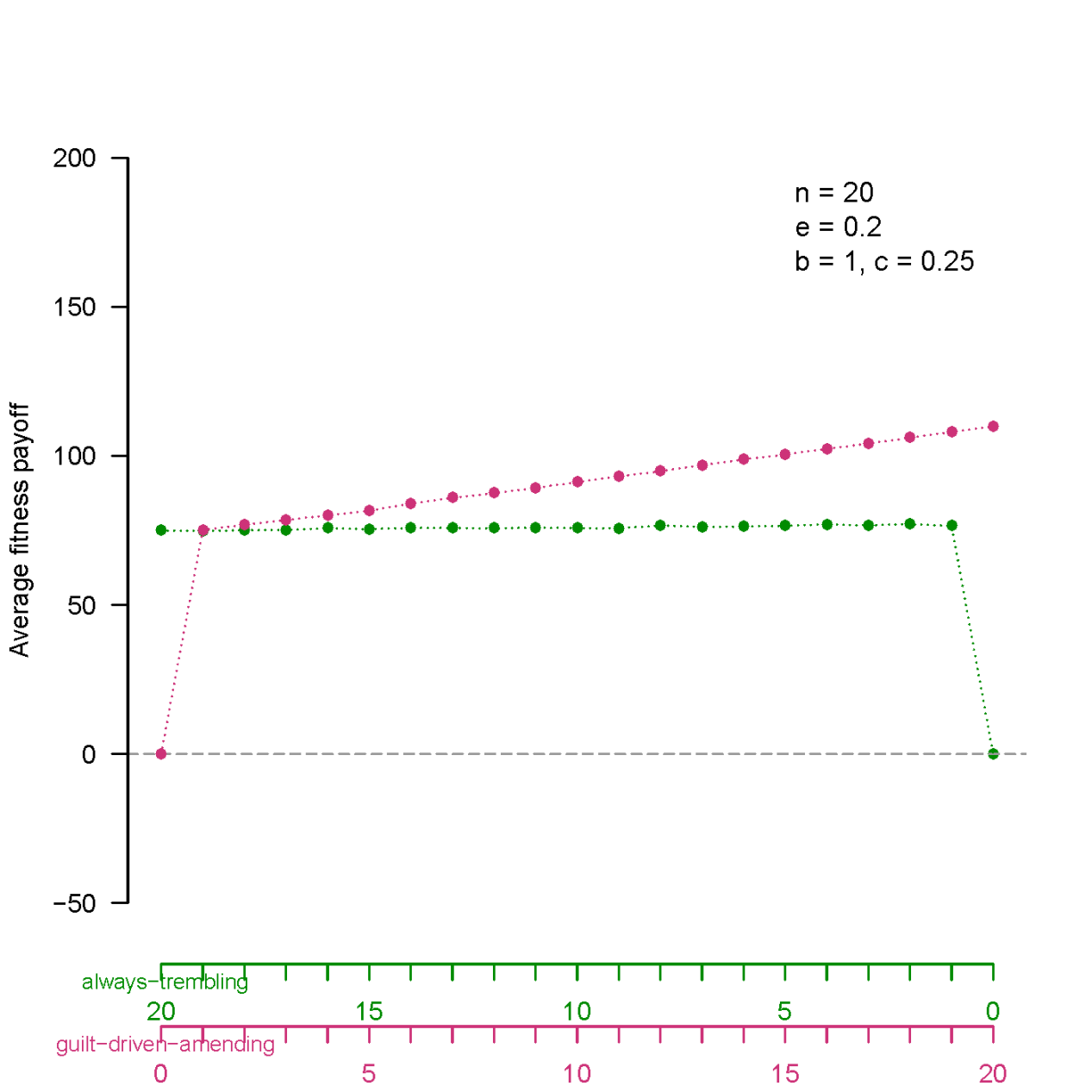


**Figure E. Always-trembling against guilt-driven-amending under the conditions that group size equals 20 (*n* = 20), benefit equals 1 and cost equals 0.25 (*b* = 1 and *c* = 0.25). Two *x* axes show that within the group the number of always-trembling individuals is decreasing from 20 to 0 and the number of guilt-driven-amending individuals is increasing from 0 to 20. The individuals from two strategies always sum up to 20. *Y* axis show the average fitness payoff for always-trembling and guilt-driven-amending. Please note that error rate (*e* = 0.2) only applies to guilt-driven-amending.**

In Figure E, we can see that one single guilt-driven-amending individual can invade a 19-always-trembling-individual group and has the same fitness payoff as always-trembling ones. At this point, the group reaches an unstable equilibrium. If the number of guilt-driven-amending individuals starts to increase, they will take over the whole group while always-trembling individual won’t be able to invade a guilt-driven-amending group.

Dynamic 6: A strategy is mono-stable to B strategy. A can invade a B group, but it will have the same payoff as B at the beginning and group is in a status of unstable equilibrium (mixed ESS) at this moment. If the number of A individuals starts to increase, the group will be finally taken over by A. B cannot invade an A group and A is a pure ESS.


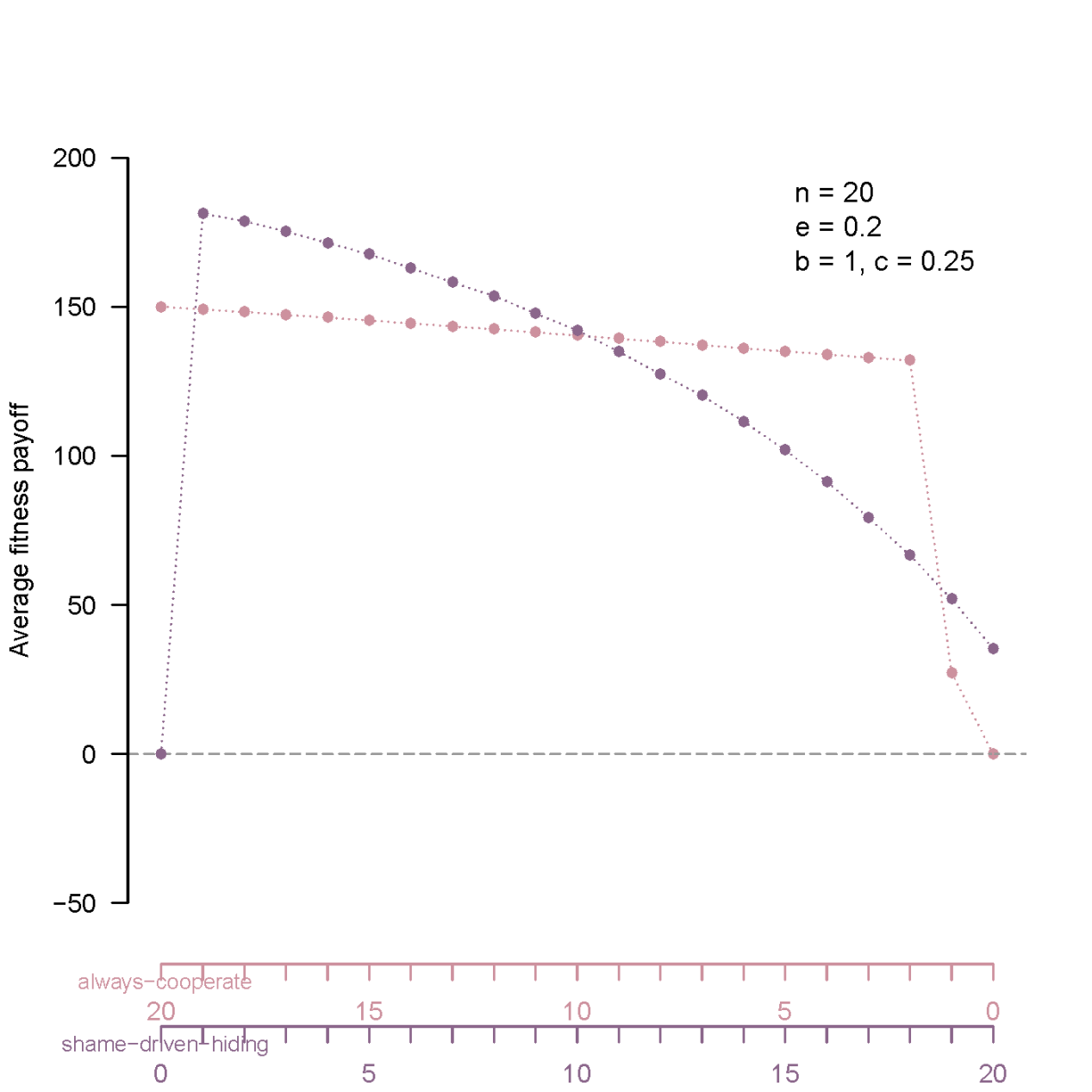


**Figure F. Always-cooperate against shame-driven-hiding under the conditions that group size equals 20 (*n* = 20), benefit equals 1 and cost equals 0.25 (*b* = 1 and *c* = 0.25). Two *x* axes show that within the group the number of always-cooperate individuals is decreasing from 20 to 0 and the number of shame-driven-hiding individuals is increasing from 0 to 20. The individuals from two strategies always sum up to 20. *Y* axis show the average fitness payoff for always-cooperate against shame-driven-hiding. Please note that error rate (*e* = 0.2) only applies to shame-driven-hiding.**

In Figure F, we can see that always-cooperate individual cannot invade a shame-driven-hiding group while shame-driven-hiding individual can invade an always-cooperate group. However, always-cooperate individuals can restrict the shame-driven-hiding individuals’ invasion in certain extent and there is a stable equilibrium point in the group. At this point, two strategies co-exist. In Figure S6, the stable equilibrium point is where the number of shame-driven-hiding individuals equals 10 and always-cooperate individuals equals 10.

Dynamic 7: A strategy is mono-stable to and co-exists with B strategy. A strategy can invade a B group while B can prevent A’s spreading to the whole group. If A invades B, there exists a stable equilibrium point where both strategies can co-exist (the group is a mixed ESS). However, B strategy cannot invade an A group. A is a pure ESS if A forms a homogeneous group.

**Table A. The summary of selection dynamics in pairwise contests.**

| **Dynamic** | **Description** |
| --- | --- |
| Dominant | A strategy dominate B strategy and A is a pure ESS. |
| Being dominated | A strategy is dominated by B strategy and B is a pure ESS. |
| Neutral | A and B strategies constitute a mixed ESS and the group is in an unstable equilibrium. |
| Co-existing | A and B strategies constitute a mixed ESS and the group is in a stable equilibrium |
| Bistable | Either A or B strategy can be a pure ESS. Which of them is the pure ESS depends on their initial frequency in the group |
| *Monostable | A strategy can invade a B group and the group is in an unstable equilibrium (mixed ESS) at first, but B cannot invade an A group and thus A is a pure ESS. |
| Monostable and co-existing | A strategy can invade a B group and co-exist with B, but B cannot invade an A group. The group is in a status of either a mixed ESS or a pure ESS (A strategy). |

*Mono-stable is a special case of bistable dynamic.

1. *The results of pairwise contests*

Under our parameter combinations, there are total 4320 pairwise contest results for ten strategies. We are unable to list so many results in this paper. For clear display of these results, we organize them into the pairwise contest tables according to different parameter combinations (please see the appendix tables for details).

**Table B. The results of pairwise contests for nine strategies when group size equals 20 (*n* = 20), benefit equals 1 (*b* = 1), cost equals 0.25 (*c* = 0.25), and error rate equals 0.2 (*e* = 0.2).**

|  | AC | AD | AT | TFT | TWTH | SDH | SDD | GDA | P | GTFT |
| --- | --- | --- | --- | --- | --- | --- | --- | --- | --- | --- |
| AC |  |  |  |  |  | SDH stable |  |  |  |  |
| AD |  |  |  |  |  |  |  |  |  |  |
| AT |  |  |  |  |  |  |  | GDA stable |  |  |
| TFT |  |  |  |  |  |  |  |  |  |  |
| TWTH |  |  |  |  |  |  |  |  |  |  |
| SDH | SDH stable |  |  |  |  |  |  |  |  | SDH stable |
| SDD |  |  |  |  |  |  |  |  |  |  |
| GDA |  |  | GDA stable |  |  |  |  |  |  |  |
| P |  |  |  |  |  |  |  |  |  |  |
| GTFT |  |  |  |  |  | SDH stable |  |  |  |  |

Note: AC stands for always-cooperate. AD stands for always-defect. At stands for always-trembling. TFT stands for tit-for-tat. TWTH stands for TFT-with-trembling-hand. SDH stands for shame-driven-hiding. SDD stands for shame-driven-hiding. GDA stands for guilt-driven-amending. P stands for Pavlov. GTFT stands for generous tit-for-tat.

The results displayed in pairwise contest table primarily refer to the row strategy (Table B). Red color **■** means that row strategy dominates column strategy. Yellow **■** means that row strategy is dominated by column strategy. Blue **■** means that row and column strategies are bistable. Green **■** means that row and column strategies are neutral. Purple **■** means that row and column strategies co-exist. Light blue **■** means that row and column strategies are monostable (which one is ESS is shown in the cell). Magenta **■** means that row and column strategies are monostable and co-existing (which one is ESS is shown in the cell).


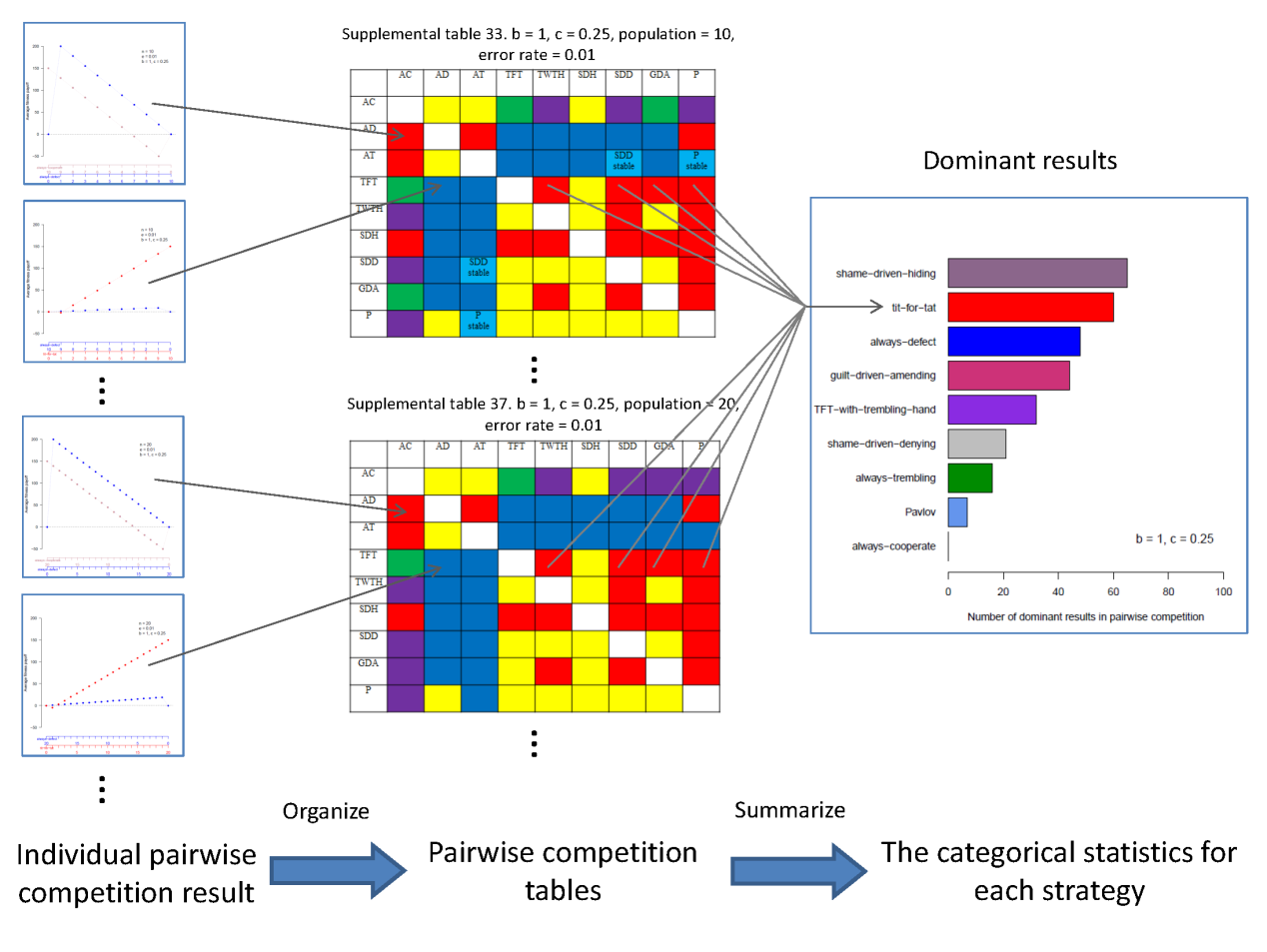


**Figure G. The workflow of organizing and summarizing pairwise contest results.**

Although we organized pairwise contest results into tables, it is still very difficult to read all these tables. In order to fully comprehend nine strategies’ performance in pairwise contest, we summarized these results into three categories according to different cost and benefit combinations (Figure G). These categories are:

1. Dominant: the number of the results in which a strategy dominates the strategies；
2. Neutral and co-existing: the number of the results in which a strategy is neutral to or co-existing with the strategies；
3. Bistable and mono-stable: the number of the results in which a strategy is bistable or mono-stable to the other strategies.


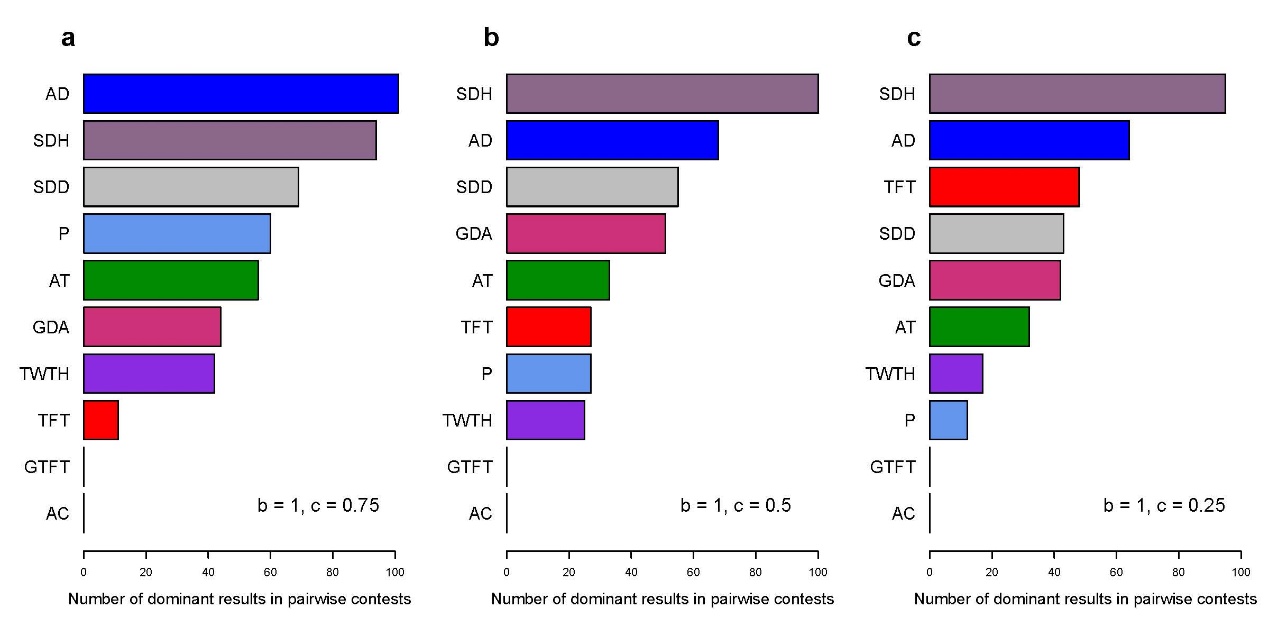


**Figure H. The number of dominant results for ten strategies in pairwise contests. (a) When benefit equals to 1 and cost equals to 0.75. (b) When benefit equals to 1 and cost equals to 0.5. (c) When benefit equals to 1 and cost equals to 0.25.**

In dominant category (Figure H), when *b* = 1 and *c* = 0.75, always defect, shame-driven-hiding and shame-driven-denying are three strategies with most dominant results; when *b* = 1 and *c* = 0.5, shame-driven-hiding, always defect and shame-driven-denying are top three strategies with most dominant results; when *b* = 1 and *c* = 0.25, shame-driven-hiding, always defect and tit-for-tat are top three strategies with most dominant results.

In this category, we can see that shame-driven-hiding has the most dominant results among nine strategies, which indicates that shame-driven-hiding is most likely to be the ESS dominating the other strategies in a group.


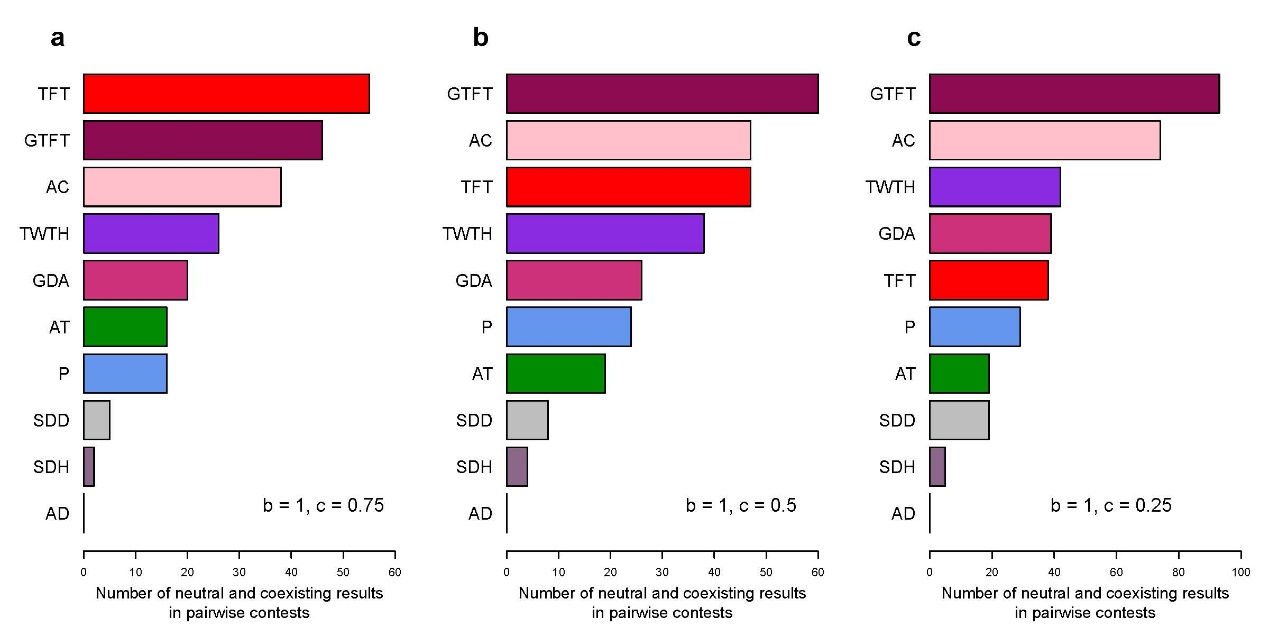


**Figure I. The number of neutral and coexisting results for ten strategies in pairwise contests. (A) When benefit equals to 1 and cost equals to 0.75. (B) When benefit equals to 1 and cost equals to 0.5. (C) When benefit equals to 1 and cost equals to 0.25.**

In neutral and co-existing category (Figure I), when *b* = 1 and *c* = 0.75, tit-for-tat, generous tit-for-tat and always-cooperate are three strategies with most neutral and co-existing results; when *b* = 1 and *c* = 0.5, generous tit-for-tat, always-cooperate and tit-for-tat are top three strategies with the neutral and co-existing results; when *b* = 1 and *c* = 0.25, generous tit-for-tat, always-cooperate and TFT-with-trembling-hand are top three strategies with the neutral and co-existing results.

In this category, we can see that generous tit-for-tat has more neutral and co-existing results than the other nine strategies, which indicates that it is most likely to be the strategy neutral to or co-existing with the other strategies in a group. Although generous tit-for-tat is most unlikely to be a pure ESS in a group, it is more likely to be the strategy that constitute a mixed ESS with the other strategies in a group. It indicates that in an unperfect world where everybody errs at some degree, forgiveness is an effective way to live with others.


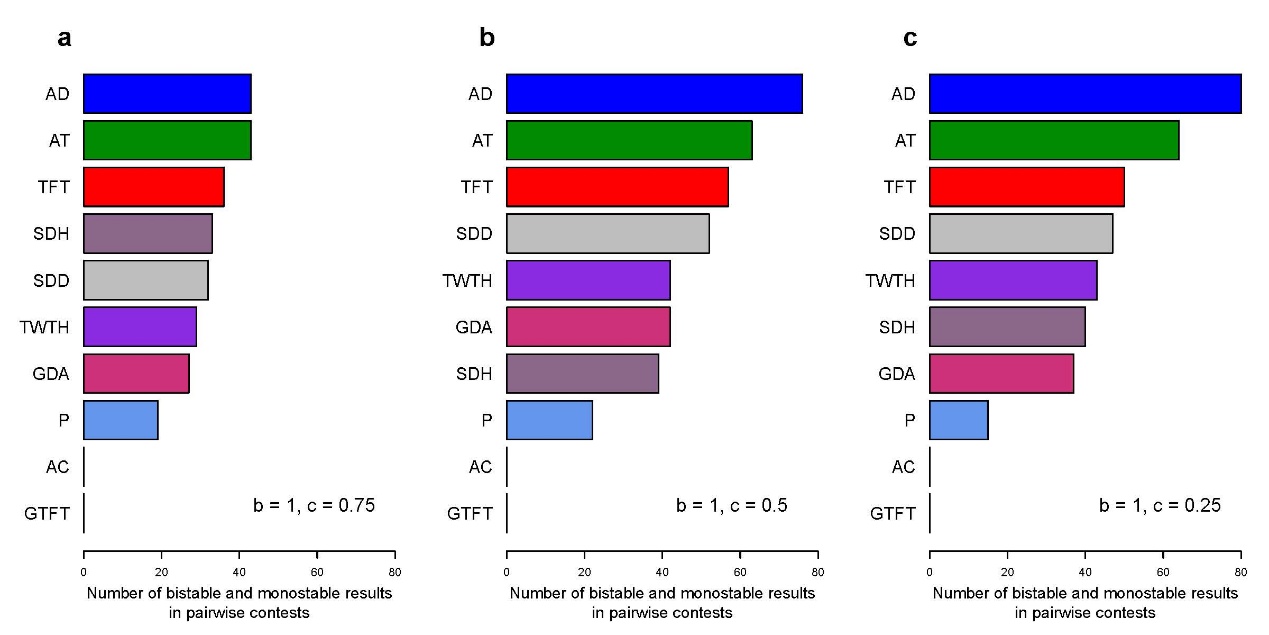


**Figure J. The number of bistable and monostable results for ten strategies in pairwise contests. (a) When benefit equals to 1 and cost equals to 0.75. (b) When benefit equals to 1 and cost equals to 0.5. (c) When benefit equals to 1 and cost equals to 0.25.**

In bistable and mono-stable category (Figure J), always-defect, always-trembling and tit-for-tat are top three strategies with most bistable and mono-stable results in all benefit and cost combinations. In this category, we can see that always-defect has the most bistable and mono-stable results among nine strategies, which indicates that always-defect is most likely to a pure ESS in a group if most of group members adopt it. This result demonstrates that the individuals with cooperation intention could hardly survived in a group mostly made of the ones who are unwilling to cooperate. Conceivably, in an always-defect group, any cooperation behavior will be met with defection and rewarded no benefit but cost. It requires at least two individuals who are truly willing to cooperate in order to survive in such a group.

Among three categories (dominant, neutral/co-existing, and bistable/mono-stable), the strategies in dominant category is capable of invading a group and taking over it. In our simulations, shame-driven-hiding is the strategy with most dominant results, which can also outperform always-defect and tit-for-tat in a group. Since human is a social species which relies on intragroup cooperation to survive and is not error free, our result proposes that self-consciousness would inevitably rise in human beings and moral emotions such as shame and guilt would be in its wake. When moral emotions granted human beings an evolutionary advantage in their social lives, they were get fixed in human population.

1. *Mathematical analysis of pairwise contests*

Assume that there exist two strategies, *I* and *J*, in a population. *E*(*I*,*J*) is the expected payoff of *I* against *J* and *E*(*J*,*I*) is the expected payoff of *J* against *I*. Since *I ≠ J* , if *I* is an ESS, then [[3](#_ENREF_3)]:

Either $E(I,I)>E(J,I)$ (1)

Or $E(I,I)=E(J,I)$ and $E(I,J)>E(J,J)$ (2)

In donation game, we assume that player A is against player B. If A has a random probability of defection *e_a_* and B has a random probability of defection *e_b_*, then the payoff matrix for A is as follows:

|  | | | Player B | |
| --- | --- | --- | --- | --- |
|  |  |  | 1*−e_b_* | *e_b_* |
|  |  |  | C | D |
| Player A | 1*−e_a_* | C | *b−c* | *−c* |
|  | *e_a_* | D | *b* | *0* |

Note: C stands for cooperation, D stands for defection, and *b* > *c* > 0.

For player A according to the matrix above, his payoff results are:

1. CC*: (1*−e_a_*)(1*−e_b_*)(*b−c*)
2. CD*: (1*−e_a_*) *e_b_*(*−c*)
3. DC*: *e_a_*(1*−e_b_*)*b*
4. DD*: *e_a_*×*e_b_*×0

*Choices are listed as A first and B second.

Let *E*(*A*,*B*) be the expected payoff of A against B*,* then *E*(*A*,*B*) = CC+CD+DC+DD = (1*−e_a_*)(1*−e_b_*)(*b−c*)+(1*−e_a_*)*e_b_*(*−c*)+*e_a_*(1*−e_b_*)*b*+*e_a_e_b_*×0 = (1*−e_b_*)*b−*(1*−e_a_*)*c*. (3)

In the same manner, we can deduce that: *E*(*B*,*A*) = (1*−e_a_*)*b−*(1*−e_b_*)*c*. (4)

From (3) and (4), we can see that in donation game each player controls cost while his opponent controls benefit.

If the number of interactions between player A and B is *T* (*T* > 0 and *T* =$\infty$), then *E*(*A*,*B*) = *T*[(1*−e_b_*)*b−*(1*−e_a_*)*c*] and *E*(*B*,*A*) = *T*[(1*−e_a_*)*b−*(1*−e_b_*)*c*].

If player A adopts the always-cooperate strategy, then *e_a_* = 0 and *E*(*A*,*B*) = *T*[(1*−e_b_*)*b−c*].

If player A adopts the always-defect strategy, then *e_a_* = 1 and *E*(*A*,*B*) = *T*[(1*−e_b_*)*b*].

If player A adopts the tit-for-tat strategy, then *e_a_* = *e_b_* and *E*(*A*,*B*) = *T*[(1*−e_b_*)*b−*(1*−e_b_*)*c*].

If player A adopts the tit-for-tat strategy while player B adopts different strategies, the mathematical analyses of their expected payoffs in pairwise contests are demonstrated as follows:

1. If player B adopts the always-cooperate or generous tit-for-tat strategy, then *e_b_* = 0. Because player A adopts the tit-for-tat strategy, then *e_a_* = *e_b_* = 0 and *E*(*A*,*B*) = *E*(*B*,*A*) = *T*(*b−c*). Thus, when tit-for-tat is against always-cooperate or generous tit-for-tat strategy, they have the same payoff and their contest result is neutral (the group is a mixed ESS).
2. If player B adopts the always-defect strategy, then *e_b_* = 1. For player A, then *e_a_* = *e_b_* = 1. However, because tit-for-tat play always cooperate in the first round, then *E*(*B*,*A*) = *b*+(*T−*1)[(1*−e_b_*)*b−*(1*−e_b_*)*c*] = *b* while *E*(*A*,*B*) = *−c*+(*T−*1)[(1*−e_b_*)*b−*(1*−e_b_*)*c*] = *−c*, and *E*(*B*,*A*) = *b* > *E*(*A*,*B*) = *−c*. Thus, in one vs. one contest, always-defect player can dominate tit-for-tat player.

However, if a tit-for-tat player is against another tit-for-tat, then *E*(*A*,*A*) = *T*(*b−c*). If *T* > *b*/(*b−c*), then *E*(*A*,*A*) > *E*(*B*,*A*) which satisfies (1). That is when *T* is large enough and there is the other tit-for-tat player in the group, tit-for-tat can be an ESS against always-defect within a group.

1. If player B adopts the TFT-with-trembling-hand strategy and defected on the *m^th^* round, then both players entered a mode of alternative retaliation and *E*(*A*,*B*) = *E*(*B*,*A*) = (*m*−*1*)(*b−c*)+(*T−m*+1)(*b*−*c*)/2, where *T* =$\infty$.

If a tit-for-tat player is against another tit-for-tat, then *E*(*A*,*A*) = *T*(*b−c*). Thus, *E*(*A*,*A*) = *T*(*b−c*) = (*m−1*)(*b−c*)+(*T−m*+*1*)(*b−c*) > *E*(*B*,*A*) = (*m−*1)(*b−c*)+(*T−m*+1)(*b−c*)/2 and tit-for-tat can be an ESS against TFT-with-trembling-hand within a group.

1. If player B adopts the always-trembling strategy and his moves are DCDCDCDC..., then *E*(*A*,*B*) = *E*(*B*,*A*) = *T*(*b*−*c*)/2, where *T* =$\infty$.

Because *E*(*A*,*A*) = *T*(*b−c*) > *E*(*B*,*A*) = *T*(*b−c*)/2 which satisfies (1), tit-for-tat can be an ESS against always-trembling within a group.

1. If player B adopts the Pavlov strategy and defected on the *m^th^* round, then *E*(*A*,*B*) = *E*(*B*,*A*) = (*m*−1)(*b*−*c*)+(*T*−*m*)(*b*−*c*)/2, where *T* =$\infty$.

Because *E*(*A*,*A*) = *T*(*b−c*) = (*m*−1)(*b*−*c*)+(*T*−*m*+1)(*b*−*c*) > *E*(*B*,*A*) = (*m*−1)(*b*−*c*)+(*T*−*m*)(*b*−*c*)/2 which satisfies (1), tit-for-tat can be an ESS against Pavlov within a group.

1. If player B adopts the guilt-driven-amending strategy and his error rate is *e_b_*, then *e_a_* = *e*_b_. Thus *E*(*B*,*A*) = *E*(*A*,*B*) =*T*[(1*−e_b_*)*b−*(1−*e_b_*)*c*] = *T*(1−*e_b_*)(*b*−*c*), where *T* =$\infty$.

Because *E*(*A*,*A*) = *T*(*b*−*c*) > *E*(*B*,*A*) = *T*(1−*e_b_*)(*b*−*c*), tit-for-tat can be an ESS against guilt-driven-amending within a group.

1. If player B adopts the shame-driven-denying strategy and defected on the *m^th^* round, then *E*(*A*,*B*) = (*m*−1)(*b*−*c*)−*c* while *E*(*B*,*A*) = (*m*−1)(*b*−*c*)+*b*. In one vs. one contest, shame-driven-denying player can dominate tit-for-tat player.

Because *E*(*A*,*A*) = *T*(*b*−*c*) = (*m*−1)(*b*−*c*) + (*T*−*m*+1)(*b*−*c*), if *T* is large enough, then (*T*−*m*+1)(*b*−*c*) > b and *E*(*A*,*A*) = (*m*−1)(*b*−*c*)+(*T*−*m*+1)(*b*−*c*) > *E*(*B*,*A*) = (*m*−1)(*b*−*c*)+*b*. That is when *T* is large enough, tit-for-tat can be an ESS against shame-driven-denying within a group.

1. If player B adopts the shame-driven-hiding strategy and is always able to hide from A’s retaliation, then *E*(*B*,*A*) = *T*[*b*−(1−*e_b_*)*c*] = *T*(*b*−*c*+*e_b_c*) while *E*(*A*,*B*) = *T*[(1−*e_b_*)*b*−*c*] = *T*(*b*−*c*−*e_b_b*). Thus *E*(*B*,*A*) > *E*(*A*,*B*).

Because *E*(*A*,*A*) = *T*(*b*−*c*), *b* > *c* > 0, and *e_b_* > 0, it is evident that *E*(*B*,*A*)= *T*(*b*−*c*+*e_b_c*) > *E*(*A*,*A*) = *T*(*b*−*c*).

If player B is against another shame-driven-hiding player and both players are always able to hide from the retaliation, then *E*(*B*,*B*) = *T*[(1−*e_b_*)*b*−(1−*e_b_*)*c*] = *T*(*b*−*c*−*e_b_b*+*e_b_c*). Thus *E*(*B*,*B*) > *E*(*A*,*B*) = *T*(*b*−*c*−*e_b_b*).

Here we can see that *E*(*B*,*A*) > *E*(*A*,*B*), *E*(*B*,*A*) > *E*(*A*,*A*), and *E*(*B*,*B*) > *E*(*A*,*B*), which satisfy both (1) and (2). Thus, shame-driven-hiding is able to dominate tit-for-tat in a group if hiding can always let shame-driven-hiding individuals evade retaliation. In another word, shame-driven-hiding is an ESS against tit-for-tat.

In the same manner, we can prove that shame-driven-hiding is an ESS against always-cooperate and generous tit-for-tat. In ii to vii, we have proved that tit-for-tat is an ESS against other eight strategies. In viii, we have also proved that shame-driven-hiding is an ESS against tit-for-tat. Thus, shame-driven-hiding can be an ESS against all the other eight strategies in our model.

The mathematical analyses above explained why shame-driven-hiding has the most dominant results in our simulation.

If player A adopts the guilt-driven-amending strategy while player B adopts the shame-driven-denying or Pavlov strategy, the mathematical analyses of their expected payoffs in pairwise contests are demonstrated as follows:

1. If player B adopts the shame-driven-denying strategy and defected on the *m^th^* round, then *E*(*A*,*B*) = (*m*−1)(*b*−*c*)−*c* while *E*(*B*,*A*) = (*m*−1)(*b*−*c*)+*b*. Because −*c* < *b*, in one vs. one contest, shame-driven-denying player can dominate guilt-driven-amending player.

However, if a guilt-driven-amending player is against another guilt-driven-amending player, their error rate is *e_a_*, then *E*(*A*,*A*) = *T*[(1−2*e_a_*)*b*−(1−2*e_a_*)*c*] = *T*[*b*−*c*−2*e_a_*(*b*−*c*)] = *T*(*b*−*c*)−2*T*×*e_a_*(*b*−*c*) = (*m*−1)(*b*−*c*)+(*T*−*m*+1)(*b*−*c*)−2*T*×*e_a_*(*b*−*c*) = (*m*−1)(*b*−*c*)+(*T*−*m*+1−2*T*×*e_a_*)(*b*−*c*).

Because *n*, *b*, *c*, and *e_a_* are fixed values while *T* =$\infty$, as long as T is large enough, we must have (*T*−*m*+1−2*T*×*e_a_*)(*b*−*c*) > *b* and thus *E*(*A*,*A*) > *E*(*B*,*A*). That is when *T* is large enough, guilt-driven-amending is an ESS against shame-driven-denying.

1. If player B adopts the Pavlov strategy and defected on the *m^th^* round, then *E*(*A*,*B*) = *E*(*B*,*A*) = (*m*−1)(*b*−*c*)+(*T*−*m*)(*b*−*c*)/2.

If a guilt-driven-amending player is against another guilt-driven-amending player, their error rate is *e_a_*, then *E*(*A*,*A*) = (*m*−1)(*b*−*c*)+(*T*−*m*+1−2*T*×*e_a_*)(*b*−*c*).

If guilt-driven-amending is an ESS against Pavlov, we must have *E*(*A*,*A*) > *E*(*B*,*A*). Therefore, (*T*−*m*+1−2*T*×*e_a_*)(*b*−*c*) must be larger than (*T*−*m*)(*b*−*c*)/2.

If (*T*−*m*+1−2*T*×*e_a_*)(*b*−*c*) > (*T*−*m*)(*b*−*c*)/2, we have 2*T*−2*m*+2−4*T*×*e_a_* > *T*−*m* which can be reduced to *T*−*m*+2 > 4*T*×*e_a_*, where *T* =$\infty$. When *T* tends to positive infinity and *m* is a fixed value, *T*−*m*+2 roughly equals to *T*. Thus, as long as *e_a_* < 0.25 (*T* > 4*T*×*e_a_*), *E*(*A*,*A*) > *E*(*B*,*A*) is satisfied.

In our simulation, the tested maximum error rate is 0.2. So in pairwise contests, guilt-driven-amending can dominate Pavlov.

At last, if a shame-driven-denying individual randomly defected on someone, he would continue to choose defection on this one in the following interactions. In this sense, shame-driven-denying is actually always-defect after it errs. Pavlov is always vulnerable to always-defect, because when it faces an always-defect opponent, it will switch back and forth between cooperation and defection[[4](#_ENREF_4)]. Thus, shame-driven-denying is an ESS against Pavlov.

1. *Multiple strategies competing in a group at the same time*

Besides pairwise contests, we also designed the simulations in which nine strategies competed against each other at the same time. Table C shows the average fitness payoff for ten strategies competing against each other in the same group under different error rates (Table C is also used as Table 2 in the main text).

**Table C. The average fitness payoff for ten strategies competing in a group under the conditions that group size is 50 (*n* = 50), benefit equals 1 and cost equals 0.25 (*b* = 1 and *c* =0.25).**

| **Strategy** | **Average fitness payoff** | | | | | |
| --- | --- | --- | --- | --- | --- | --- |
| n = 50 | e = 0.01 | e = 0.05 | e = 0.1 | e = 0.2 | e = 0.3 | e = 0.4 |
| AC (n_AC_=5) | 117.404 | 110.788 | 103.612 | 90.068 | 81.006 | 72.498 |
| AD (n_AD_=5) | 83.232 | 83.312 | 83.17 | 82.274 | 81.618 | 81.268 |
| AT  (n_AT_=5) | 97.628 | 95.851 | 93.96 | 89.362 | 84.803 | 80.479 |
| TFT (n_TFT_=5) | **123.608** | **117.078** | **110.562** | **99.483** | **91.044** | **85.198** |
| GTFT  (n_GTFT_=5) | 118.972 | 112.194 | 105.212 | 92.772 | 82.834 | 75.121 |
| TWTH (n_TWTH_=5) | 122.524 | 114.576 | 106.254 | 95.685 | **89.291** | **83.529** |
| SDH (n_SDH_=5) | **123.592** | **117.873** | **111.192** | **98.136** | 86.547 | 76.087 |
| SDD (n_SDD_=5) | 122.459 | 113.679 | 105.273 | 94.249 | 87.894 | 83.104 |
| GDA (n_GDA_=5) | **122.813** | **115.017** | **107.288** | **97.381** | **90.564** | **85.285** |
| P  (n_P_=5) | 97.999 | 96.521 | 93.662 | 89.420 | 84.771 | 80.705 |

Note: Every strategy has five individual in this group, e.g. n_AC_=5. The average fitness payoff in this table is based on 100 simulations. AC stands for always-cooperate. AD stands for always-defect. AT stands for always-trembling. TFT stands for tit-for-tat. GTFT stands for generous-TFT. TWTH stands for TFT-with-trembling-hand. SDH stands for shame-driven-hiding. SDD stands for shame-driven-denying. GDA stands for guilt-driven-amending. P stands for Pavlov. Under the same error rate, red color indicates the highest fitness payoff, blue indicates the second highest, and green indicates the third highest.

In Table C, we can see when error rate is relatively small (0.01, 0.05, 0.1 and 0.2), shame-driven hiding or tit-for-tat has the highest average fitness payoff among ten strategies and thus is the ESS in the group; while guilt-driven amending has the third highest average fitness payoff. However, when error rate is relatively large (0.3 and 0.4), guilt-driven amending or tit-for-tat becomes the strategy with the highest average fitness payoff and the ESS in the group. When error rate is 0.3 or 0.4, guilt-driven-amending outperforms shame-driven-hiding. In our model, tit-for-tat doesn’t err while to err is a part of human’s nature. Among the strategies that do err, we can see that their performance in simulations varies according to different error rates. When error rate equals to 0.01, tit-for-tat and its derivative strategies (TWTH, SDH, SDD, and GDA) have similar fitness pay-off. Thus, under this error rate, the group made of these strategies is nearly in a state of mixed equilibrium.

In pairwise contests, we found no strategy able to be an ESS against the other strategies under all parameter combinations. The result of multiple strategies competing in a group also shows that different strategy has different average fitness payoff under different error rates. In real world, the conditions are much more complicated than what we simulated in our model. Thus, based on our results, we propose that human population exhibit a mixed strategy equilibrium as far as mentality is concerned, which is in accordance with the Bishop-Cannings theorem [[5](#_ENREF_5)].

1. *The group’s average fitness payoffs of five error-prone strategies under different benefit to cost ratios*

Besides *b* = 1 and *c* = 0.25, we evaluated the group’s average fitness payoffs of five error-prone strategies under the benefit to cost ratios of 4/3 (*b* = 1 and *c* = 0.75) and 2 (*b* = 1 and *c* = 0.5). In Figure Ka and Kb, we can see that when group size is small (*n* = 10 and *n* = 20), guilt-driven-amending group has the highest average fitness payoff at any error rate; when group size is large (*n* = 50 and *n* = 100, Figure Kc and Kd), both shame-driven-hiding and Pavlov groups outperform the guilt-driven-amending group; especially when *n* = 100, the Pavlov group has the highest average fitness payoff at any error rate. However, if *b*/*c* = 4/3, when group size is larger than 50, the cooperative behavior is no longer sustainable within a group, because the ratio of defection payoff to cooperation payoff is not smaller than the number of average interaction between any two group members (*b*/(*b-c*) ≥ *T*/*n*). Thus, when *n* = 100 and *b*/*c* = 4/3, always-defect prevails in a group.

Figure K show that the group’s average fitness payoffs of five error-prone strategies under the benefit to cost ratio of 2. Under this benefit to cost ratio, the performance of five error-prone strategies is very similar their performance under the benefit to cost ratio of 4 (*b* = 1 and *c* = 0.25, the result is shown in main text). Thus, we don’t verbosely restate the result.


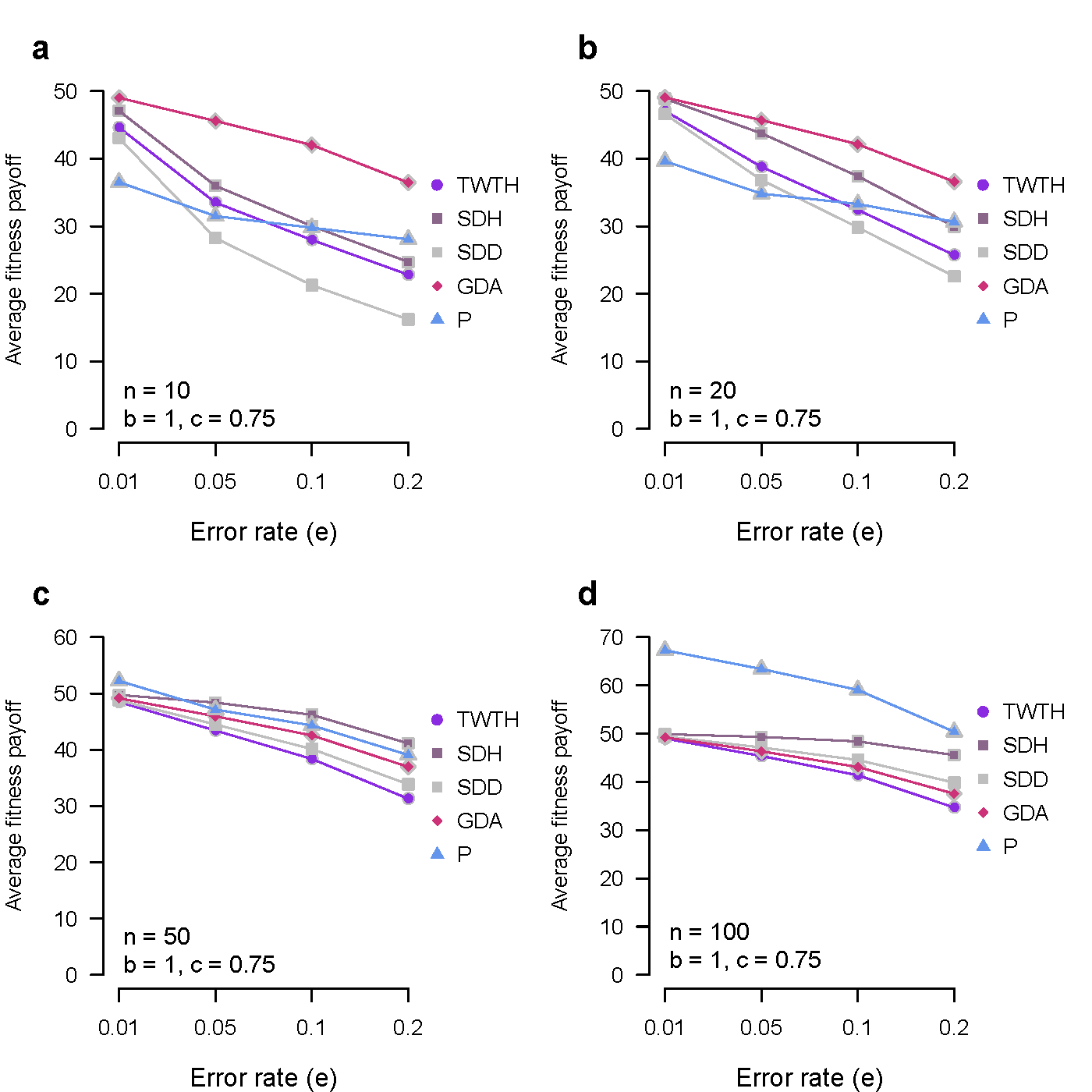


**Figure K. At four different error rates, the average fitness payoff for five homogeneous groups which adopt the error-prone strategies. TWTH stands for TFT-with-trembling-hand. SDH stands for shame-driven-hiding. SDD stands for shame-driven-denying. GDA stands for guilt-driven-amending. P stands for Pavlov. (a) When *b* = 1, *c* = 0.25, and *n* = 10. (b) When *b* = 1, *c* = 0.25, and *n* = 20. (c) When *b* = 1, *c* = 0.25, and *n* = 50. (d) When *b* = 1, *c* = 0.25, and *n* = 100.**


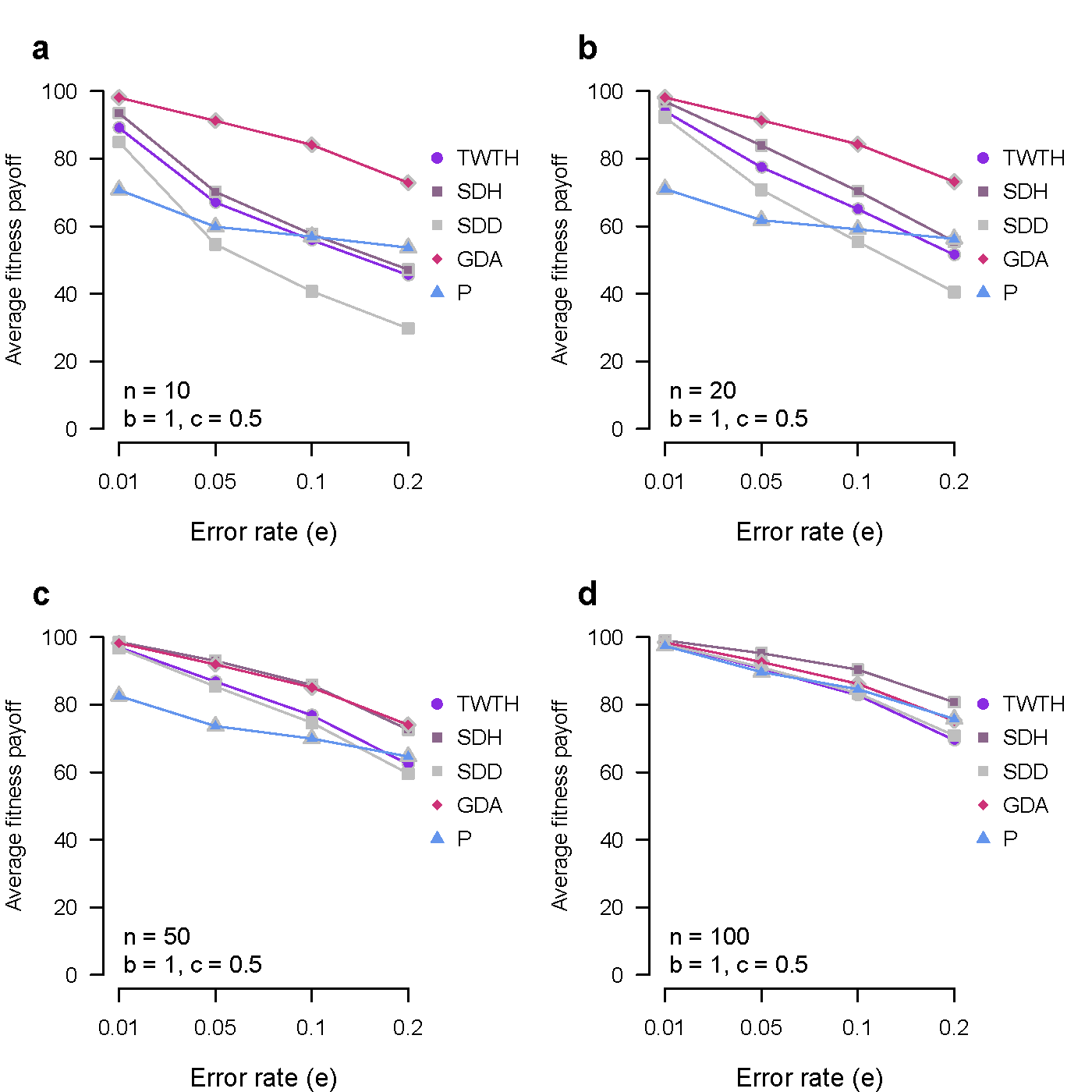


**Figure L. At four different error rates, the average fitness payoff for five homogeneous groups which adopt the error-prone strategies. TWTH stands for TFT-with-trembling-hand. SDH stands for shame-driven-hiding. SDD stands for shame-driven-denying. GDA stands for guilt-driven-amending. P stands for Pavlov. (a) When *b* = 1, *c* = 0.25, and *n* = 10. (b) When *b* = 1, *c* = 0.25, and *n* = 20. (c) When *b* = 1, *c* = 0.25, and *n* = 50. (d) When *b* = 1, *c* = 0.25, and *n* = 100.**

1. *Source code and contest results*

The computer simulation for studying the evolution of shame and guilt is written in Perl programing language. The Perl source codes can be downloaded from <https://github.com/libing-shen/The-evolution-of-shame-and-guilt>, which also includes the pairwise contest results and result summaries.

1. *References*

1. Axelrod R, Hamilton WD (1981) The evolution of cooperation. Science 211: 1390-1396.

2. Nowak MA (2006) Evolutionary dynamics : exploring the equations of life. Cambridge, Mass.: Belknap Press of Harvard University Press. xi, 363 p. p.

3. Maynard Smith J (1982) Evolution and the theory of games. Cambridge ; New York: Cambridge University Press. viii, 224 p. p.

4. Nowak M, Sigmund K (1993) A strategy of win-stay, lose-shift that outperforms tit-for-tat in the Prisoner's Dilemma game. Nature 364: 56-58.

5. Bishop DT, Cannings C (1978) A generalized war of attrition. J Theor Biol 70: 85-124.

1. *Appendix: pairwise-contest results organized in table format*

Appendix table 1. Pairwise contest results for 9 strategies when b =1, c = 0.75, n = 10, e = 0.01. ***Please see section 4 (*The results of pairwise contests*) for the meaning of each color.***

|  | AC | AD | AT | TFT | TWTH | SDH | SDD | GDA | P |
| --- | --- | --- | --- | --- | --- | --- | --- | --- | --- |
| AC |  |  |  |  |  |  |  |  |  |
| AD |  |  |  |  |  |  |  |  |  |
| AT |  |  |  |  |  |  |  |  |  |
| TFT |  |  |  |  |  |  |  |  |  |
| TWTH |  |  |  |  |  |  |  |  |  |
| SDH |  |  |  |  |  |  |  |  |  |
| SDD |  |  |  |  |  |  |  |  |  |
| GDA |  |  |  |  |  |  |  |  |  |
| P |  |  |  |  |  |  |  |  |  |

Appendix table 2. Pairwise contest results for 9 strategies when b =1, c = 0.75, n = 10, e = 0.05.

|  | AC | AD | AT | TFT | TWTH | SDH | SDD | GDA | P |
| --- | --- | --- | --- | --- | --- | --- | --- | --- | --- |
| AC |  |  |  |  |  |  |  |  |  |
| AD |  |  |  |  |  |  |  |  |  |
| AT |  |  |  |  |  | SDH stable |  |  |  |
| TFT |  |  |  |  |  |  |  |  |  |
| TWTH |  |  |  |  |  |  |  |  |  |
| SDH |  |  | SDH stable |  |  |  |  |  |  |
| SDD |  |  |  |  |  |  |  |  |  |
| GDA |  |  |  |  |  |  |  |  |  |
| P |  |  |  |  |  |  |  |  |  |

Appendix table 3. Pairwise contest results for 9 strategies when b =1, c = 0.75, n = 10, e = 0.1.

|  | AC | AD | AT | TFT | TWTH | SDH | SDD | GDA | P |
| --- | --- | --- | --- | --- | --- | --- | --- | --- | --- |
| AC |  |  |  |  |  |  |  |  |  |
| AD |  |  |  |  |  | AD stable | AD stable |  |  |
| AT |  |  |  |  | TWTH stable |  |  |  |  |
| TFT |  |  |  |  | TFT stable | TFT stable |  |  |  |
| TWTH |  |  | TWTH stable | TFT stable |  |  |  | GDA stable |  |
| SDH |  | AD stable |  | TFT stable |  |  |  |  |  |
| SDD |  | AD stable |  |  |  |  |  | GDA stable |  |
| GDA |  |  |  |  | GDA stable |  | GDA stable |  |  |
| P |  |  |  |  |  |  |  |  |  |

Appendix table 4. Pairwise contest results for 9 strategies when b =1, c = 0.75, n = 10, e = 0.2.

|  | AC | AD | AT | TFT | TWTH | SDH | SDD | GDA | P |
| --- | --- | --- | --- | --- | --- | --- | --- | --- | --- |
| AC |  |  |  |  |  |  |  |  |  |
| AD |  |  |  |  |  |  |  |  |  |
| AT |  |  |  |  |  |  |  |  |  |
| TFT |  |  |  |  |  |  |  |  |  |
| TWTH |  |  |  |  |  |  |  |  |  |
| SDH |  |  |  |  |  |  |  |  |  |
| SDD |  |  |  |  |  |  |  |  |  |
| GDA |  |  |  |  |  |  |  |  |  |
| P |  |  |  |  |  |  |  |  |  |

Appendix table 5. Pairwise contest results for 9 strategies when b =1, c = 0.75, n = 20, e = 0.01.

|  | AC | AD | AT | TFT | TWTH | SDH | SDD | GDA | P |
| --- | --- | --- | --- | --- | --- | --- | --- | --- | --- |
| AC |  |  |  |  | TWTH stable |  |  |  |  |
| AD |  |  |  |  |  |  |  |  |  |
| AT |  |  |  |  |  |  |  |  |  |
| TFT |  |  |  |  |  |  | TFT stable |  |  |
| TWTH | TWTH stable |  |  |  |  |  |  |  |  |
| SDH |  |  |  |  |  |  |  |  |  |
| SDD |  |  |  | TFT stable |  |  |  |  |  |
| GDA |  |  |  |  |  |  |  |  |  |
| P |  |  |  |  |  |  |  |  |  |

Appendix table 6. Pairwise contest results for 9 strategies when b =1, c = 0.75, n = 20, e = 0.05.

|  | AC | AD | AT | TFT | TWTH | SDH | SDD | GDA | P |
| --- | --- | --- | --- | --- | --- | --- | --- | --- | --- |
| AC |  |  |  |  | TWTH stable |  |  |  |  |
| AD |  |  |  |  |  |  |  |  |  |
| AT |  |  |  |  |  |  |  |  |  |
| TFT |  |  |  |  | TFT stable |  |  |  |  |
| TWTH | TWTH stable |  |  | TFT stable |  |  |  |  |  |
| SDH |  |  |  |  |  |  |  |  |  |
| SDD |  |  |  |  |  |  |  | GDA stable |  |
| GDA |  |  |  |  |  |  | GDA stable |  |  |
| P |  |  |  |  |  |  |  |  |  |

Appendix table 7. Pairwise contest results for 9 strategies when b =1, c = 0.75, n = 20, e = 0.1.

|  | AC | AD | AT | TFT | TWTH | SDH | SDD | GDA | P |
| --- | --- | --- | --- | --- | --- | --- | --- | --- | --- |
| AC |  |  |  |  | TWTH stable |  |  |  |  |
| AD |  |  |  |  |  |  | AD stable |  |  |
| AT |  |  |  |  |  | SDH stable |  |  |  |
| TFT |  |  |  |  | TFT stable |  |  |  |  |
| TWTH | TWTH stable |  |  | TFT stable |  |  |  |  | TWTH stable |
| SDH |  |  | SDH stable |  |  |  |  |  |  |
| SDD |  | AD stable |  |  |  |  |  |  |  |
| GDA |  |  |  |  |  |  |  |  |  |
| P |  |  |  |  | TWTH stable |  |  |  |  |

Appendix table 8. Pairwise contest results for 9 strategies when b =1, c = 0.75, n = 20, e = 0.2.

|  | AC | AD | AT | TFT | TWTH | SDH | SDD | GDA | P |
| --- | --- | --- | --- | --- | --- | --- | --- | --- | --- |
| AC |  |  |  |  |  |  |  |  |  |
| AD |  |  |  |  |  |  |  |  |  |
| AT |  |  |  |  |  |  |  |  |  |
| TFT |  |  |  |  |  |  |  |  |  |
| TWTH |  |  |  |  |  |  |  | TWTH stable |  |
| SDH |  |  |  |  |  |  |  |  |  |
| SDD |  |  |  |  |  |  |  |  |  |
| GDA |  |  |  |  | TWTH stable |  |  |  |  |
| P |  |  |  |  |  |  |  |  |  |

Appendix table 9. Pairwise contest results for 9 strategies when b =1, c = 0.75, n = 50, e = 0.01.

|  | AC | AD | AT | TFT | TWTH | SDH | SDD | GDA | P |
| --- | --- | --- | --- | --- | --- | --- | --- | --- | --- |
| AC |  |  |  |  |  |  |  |  |  |
| AD |  |  |  | AD stable |  | AD stable |  | AD stable |  |
| AT |  |  |  | AT stable | AT stable |  | AT stable | AT stable | P stable |
| TFT |  | AD stable | AT stable |  |  |  |  |  |  |
| TWTH |  |  | AT stable |  |  |  |  |  |  |
| SDH |  | AD stable |  |  |  |  |  |  |  |
| SDD |  |  | AT stable |  |  |  |  |  |  |
| GDA |  | AD stable | AT stable |  |  |  |  |  |  |
| P |  |  | P stable |  |  |  |  |  |  |

Appendix table 10. Pairwise contest results for 9 strategies when b =1, c = 0.75, n = 50, e = 0.05.

|  | AC | AD | AT | TFT | TWTH | SDH | SDD | GDA | P |
| --- | --- | --- | --- | --- | --- | --- | --- | --- | --- |
| AC |  |  |  |  |  |  |  |  |  |
| AD |  |  |  | AD stable |  |  |  |  |  |
| AT |  |  |  | AT stable |  |  |  |  |  |
| TFT |  | AD stable | AT stable |  |  |  |  |  |  |
| TWTH |  |  |  |  |  |  |  |  |  |
| SDH |  |  |  |  |  |  |  |  |  |
| SDD |  |  |  |  |  |  |  |  |  |
| GDA |  |  |  |  |  |  |  |  |  |
| P |  |  |  |  |  |  |  |  |  |

Appendix table 11. Pairwise contest results for 9 strategies when b =1, c = 0.75, n = 50, e = 0.1.

|  | AC | AD | AT | TFT | TWTH | SDH | SDD | GDA | P |
| --- | --- | --- | --- | --- | --- | --- | --- | --- | --- |
| AC |  |  |  |  |  |  |  |  |  |
| AD |  |  |  | AD stable |  |  |  |  |  |
| AT |  |  |  | AT stable |  |  |  |  |  |
| TFT |  | AD stable | AT stable |  |  |  |  |  | P stable |
| TWTH |  |  |  |  |  |  |  |  |  |
| SDH |  |  |  |  |  |  |  |  | SDH stable |
| SDD |  |  |  |  |  |  |  |  |  |
| GDA |  |  |  |  |  |  |  |  |  |
| P |  |  |  | P stable |  | SDH stable |  |  |  |

Appendix table 12. Pairwise contest results for 9 strategies when b =1, c = 0.75, n = 50, e = 0.2.

|  | AC | AD | AT | TFT | TWTH | SDH | SDD | GDA | P |
| --- | --- | --- | --- | --- | --- | --- | --- | --- | --- |
| AC |  |  |  |  |  |  |  |  |  |
| AD |  |  |  | AD stable |  |  |  |  |  |
| AT |  |  |  | AT stable |  |  |  |  |  |
| TFT |  | AD stable | AT stable |  |  |  |  | GDA stable | P stable |
| TWTH |  |  |  |  |  |  |  |  |  |
| SDH |  |  |  |  |  |  |  |  |  |
| SDD |  |  |  |  |  |  |  |  |  |
| GDA |  |  |  | GDA stable |  |  |  |  |  |
| P |  |  |  | P stable |  |  |  |  |  |

Appendix table 13. Pairwise contest results for 9 strategies when b =1, c = 0.75, n = 100, e = 0.01.

|  | AC | AD | AT | TFT | TWTH | SDH | SDD | GDA | P |
| --- | --- | --- | --- | --- | --- | --- | --- | --- | --- |
| AC |  |  |  |  |  |  |  |  |  |
| AD |  |  |  |  |  |  |  |  |  |
| AT |  |  |  |  |  |  |  |  |  |
| TFT |  |  |  |  |  |  |  |  |  |
| TWTH |  |  |  |  |  |  |  |  |  |
| SDH |  |  |  |  |  |  |  |  |  |
| SDD |  |  |  |  |  |  |  |  |  |
| GDA |  |  |  |  |  |  |  |  |  |
| P |  |  |  |  |  |  |  |  |  |

Appendix table 14. Pairwise contest results for 9 strategies when b =1, c = 0.75, n = 100, e = 0.05.

|  | AC | AD | AT | TFT | TWTH | SDH | SDD | GDA | P |
| --- | --- | --- | --- | --- | --- | --- | --- | --- | --- |
| AC |  |  |  |  |  |  |  |  |  |
| AD |  |  |  |  |  |  |  |  |  |
| AT |  |  |  |  |  |  |  |  |  |
| TFT |  |  |  |  |  |  |  |  |  |
| TWTH |  |  |  |  |  |  |  |  |  |
| SDH |  |  |  |  |  |  |  |  |  |
| SDD |  |  |  |  |  |  |  |  |  |
| GDA |  |  |  |  |  |  |  |  |  |
| P |  |  |  |  |  |  |  |  |  |

Appendix table 15. Pairwise contest results for 9 strategies when b =1, c = 0.75, n = 100, e = 0.1.

|  | AC | AD | AT | TFT | TWTH | SDH | SDD | GDA | P |
| --- | --- | --- | --- | --- | --- | --- | --- | --- | --- |
| AC |  |  |  |  |  |  |  |  |  |
| AD |  |  |  |  |  |  |  |  |  |
| AT |  |  |  |  |  |  |  |  |  |
| TFT |  |  |  |  |  |  |  |  |  |
| TWTH |  |  |  |  |  |  |  |  |  |
| SDH |  |  |  |  |  |  |  |  |  |
| SDD |  |  |  |  |  |  |  |  |  |
| GDA |  |  |  |  |  |  |  |  |  |
| P |  |  |  |  |  |  |  |  |  |

Appendix table 16. Pairwise contest results for 9 strategies when b =1, c = 0.75, n = 100, e = 0.2.

|  | AC | AD | AT | TFT | TWTH | SDH | SDD | GDA | P |
| --- | --- | --- | --- | --- | --- | --- | --- | --- | --- |
| AC |  |  |  |  |  |  |  |  |  |
| AD |  |  |  |  |  |  |  |  |  |
| AT |  |  |  |  |  |  |  |  |  |
| TFT |  |  |  |  |  |  |  |  |  |
| TWTH |  |  |  |  |  |  |  |  |  |
| SDH |  |  |  |  |  |  |  |  |  |
| SDD |  |  |  |  |  |  |  |  |  |
| GDA |  |  |  |  |  |  |  |  |  |
| P |  |  |  |  |  |  |  |  |  |

Appendix table 17. Pairwise contest results for 9 strategies when b =1, c = 0.5, n = 10, e = 0.01.

|  | AC | AD | AT | TFT | TWTH | SDH | SDD | GDA | P |
| --- | --- | --- | --- | --- | --- | --- | --- | --- | --- |
| AC |  |  |  |  |  |  |  | GDA stable |  |
| AD |  |  |  |  |  |  |  |  |  |
| AT |  |  |  |  |  |  | SDD stable |  | P stable |
| TFT |  |  |  |  |  |  |  |  |  |
| TWTH |  |  |  |  |  |  |  |  |  |
| SDH |  |  |  |  |  |  |  |  |  |
| SDD |  |  | SDD stable |  |  |  |  |  |  |
| GDA | GDA stable |  |  |  |  |  |  |  |  |
| P |  |  | P stable |  |  |  |  |  |  |

Appendix table 18. Pairwise contest results for 9 strategies when b =1, c = 0.5, n = 10, e = 0.05.

|  | AC | AD | AT | TFT | TWTH | SDH | SDD | GDA | P |
| --- | --- | --- | --- | --- | --- | --- | --- | --- | --- |
| AC |  |  |  |  |  | SDH stable |  |  |  |
| AD |  |  |  |  |  |  |  |  |  |
| AT |  |  |  |  |  | SDH stable |  |  | P stable |
| TFT |  |  |  |  |  |  |  |  |  |
| TWTH |  |  |  |  |  |  |  |  |  |
| SDH | SDH stable |  | SDH stable |  |  |  |  |  |  |
| SDD |  |  |  |  |  |  |  |  |  |
| GDA |  |  |  |  |  |  |  |  | GDA stable |
| P |  |  | P stable |  |  |  |  | GDA stable |  |

Appendix table 19. Pairwise contest results for 9 strategies when b =1, c = 0.5, n = 10, e = 0.1.

|  | AC | AD | AT | TFT | TWTH | SDH | SDD | GDA | P |
| --- | --- | --- | --- | --- | --- | --- | --- | --- | --- |
| AC |  |  |  |  |  | SDH stable |  |  |  |
| AD |  |  |  |  |  |  |  |  |  |
| AT |  |  |  |  | TWTH stable |  |  | GDA stable | P stable |
| TFT |  |  |  |  |  |  |  |  |  |
| TWTH |  |  | TWTH stable |  |  |  |  |  |  |
| SDH | SDH stable |  |  |  |  |  |  |  |  |
| SDD |  |  |  |  |  |  |  |  |  |
| GDA |  |  | GDA stable |  |  |  |  |  |  |
| P |  |  | P stable |  |  |  |  |  |  |

Appendix table 20. Pairwise contest results for 9 strategies when b =1, c = 0.5, n = 10, e = 0.2.

|  | AC | AD | AT | TFT | TWTH | SDH | SDD | GDA | P |
| --- | --- | --- | --- | --- | --- | --- | --- | --- | --- |
| AC |  |  |  |  |  | SDH stable |  |  |  |
| AD |  |  |  |  |  |  |  |  |  |
| AT |  |  |  |  |  |  |  |  |  |
| TFT |  |  |  |  |  |  |  |  |  |
| TWTH |  |  |  |  |  |  |  |  |  |
| SDH | SDH stable |  |  |  |  |  |  |  |  |
| SDD |  |  |  |  |  |  |  |  |  |
| GDA |  |  |  |  |  |  |  |  |  |
| P |  |  |  |  |  |  |  |  |  |

Appendix table 21. Pairwise contest results for 9 strategies when b =1, c = 0.5, n = 20, e = 0.01.

|  | AC | AD | AT | TFT | TWTH | SDH | SDD | GDA | P |
| --- | --- | --- | --- | --- | --- | --- | --- | --- | --- |
| AC |  |  |  |  |  |  |  | GDA stable |  |
| AD |  |  |  |  |  |  |  |  |  |
| AT |  |  |  |  |  |  |  |  |  |
| TFT |  |  |  |  |  |  | TFT stable |  |  |
| TWTH |  |  |  |  |  |  |  |  |  |
| SDH |  |  |  |  |  |  |  |  |  |
| SDD |  |  |  | TFT stable |  |  |  |  |  |
| GDA | GDA stable |  |  |  |  |  |  |  |  |
| P |  |  |  |  |  |  |  |  |  |

Appendix table 22. Pairwise contest results for 9 strategies when b =1, c = 0.5, n = 20, e = 0.05.

|  | AC | AD | AT | TFT | TWTH | SDH | SDD | GDA | P |
| --- | --- | --- | --- | --- | --- | --- | --- | --- | --- |
| AC |  |  |  |  |  |  |  |  |  |
| AD |  |  |  |  |  |  |  |  |  |
| AT |  |  |  |  |  |  |  |  |  |
| TFT |  |  |  |  |  |  |  |  |  |
| TWTH |  |  |  |  |  |  |  |  |  |
| SDH |  |  |  |  |  |  |  |  |  |
| SDD |  |  |  |  |  |  |  |  |  |
| GDA |  |  |  |  |  |  |  |  |  |
| P |  |  |  |  |  |  |  |  |  |

Appendix table 23. Pairwise contest results for 9 strategies when b =1, c = 0.5, n = 20, e = 0.1.

|  | AC | AD | AT | TFT | TWTH | SDH | SDD | GDA | P |
| --- | --- | --- | --- | --- | --- | --- | --- | --- | --- |
| AC |  |  |  |  |  |  |  |  |  |
| AD |  |  |  |  |  |  |  |  |  |
| AT |  |  |  |  |  | SDH stable |  |  |  |
| TFT |  |  |  |  |  |  |  |  |  |
| TWTH |  |  |  |  |  |  |  |  |  |
| SDH |  |  | SDH stable |  |  |  |  |  |  |
| SDD |  |  |  |  |  |  |  |  |  |
| GDA |  |  |  |  |  |  |  |  | GDA stable |
| P |  |  |  |  |  |  |  | GDA stable |  |

Appendix table 24. Pairwise contest results for 9 strategies when b =1, c = 0.5, n = 20, e = 0.2.

|  | AC | AD | AT | TFT | TWTH | SDH | SDD | GDA | P |
| --- | --- | --- | --- | --- | --- | --- | --- | --- | --- |
| AC |  |  |  |  |  | SDH stable |  |  |  |
| AD |  |  |  |  |  |  |  |  |  |
| AT |  |  |  |  |  |  |  | GDA stable |  |
| TFT |  |  |  |  |  |  | TFT stable |  |  |
| TWTH |  |  |  |  |  |  |  |  |  |
| SDH | SDH stable |  |  |  |  |  |  |  |  |
| SDD |  |  |  | TFT stable |  |  |  |  |  |
| GDA |  |  | GDA stable |  |  |  |  |  |  |
| P |  |  |  |  |  |  |  |  |  |

Appendix table 25. Pairwise contest results for 9 strategies when b =1, c = 0.5, n = 50, e = 0.01.

|  | AC | AD | AT | TFT | TWTH | SDH | SDD | GDA | P |
| --- | --- | --- | --- | --- | --- | --- | --- | --- | --- |
| AC |  |  |  |  |  |  |  | GDA stable |  |
| AD |  |  |  |  |  |  |  |  |  |
| AT |  |  |  |  |  |  |  |  | P stable |
| TFT |  |  |  |  |  |  |  |  |  |
| TWTH |  |  |  |  |  |  |  |  |  |
| SDH |  |  |  |  |  |  |  |  |  |
| SDD |  |  |  |  |  |  |  |  |  |
| GDA | GDA stable |  |  |  |  |  |  |  |  |
| P |  |  | P stable |  |  |  |  |  |  |

Appendix table 26. Pairwise contest results for 9 strategies when b =1, c = 0.5, n = 50, e = 0.05.

|  | AC | AD | AT | TFT | TWTH | SDH | SDD | GDA | P |
| --- | --- | --- | --- | --- | --- | --- | --- | --- | --- |
| AC |  |  |  |  |  |  |  |  |  |
| AD |  |  |  |  |  |  |  |  |  |
| AT |  |  |  |  |  |  |  |  |  |
| TFT |  |  |  |  |  |  |  |  |  |
| TWTH |  |  |  |  |  |  |  |  |  |
| SDH |  |  |  |  |  |  |  |  |  |
| SDD |  |  |  |  |  |  |  |  |  |
| GDA |  |  |  |  |  |  |  |  |  |
| P |  |  |  |  |  |  |  |  |  |

Appendix table 27. Pairwise contest results for 9 strategies when b =1, c = 0.5, n = 50, e = 0.1.

|  | AC | AD | AT | TFT | TWTH | SDH | SDD | GDA | P |
| --- | --- | --- | --- | --- | --- | --- | --- | --- | --- |
| AC |  |  |  |  |  |  |  |  |  |
| AD |  |  |  |  |  |  |  |  |  |
| AT |  |  |  |  |  |  |  |  |  |
| TFT |  |  |  |  |  |  |  |  |  |
| TWTH |  |  |  |  |  |  |  |  |  |
| SDH |  |  |  |  |  |  |  |  |  |
| SDD |  |  |  |  |  |  |  |  |  |
| GDA |  |  |  |  |  |  |  |  |  |
| P |  |  |  |  |  |  |  |  |  |

Appendix table 28. Pairwise contest results for 9 strategies when b =1, c = 0.5, n = 50, e = 0.2.

|  | AC | AD | AT | TFT | TWTH | SDH | SDD | GDA | P |
| --- | --- | --- | --- | --- | --- | --- | --- | --- | --- |
| AC |  |  |  |  |  |  |  |  |  |
| AD |  |  |  |  |  |  |  |  |  |
| AT |  |  |  |  |  |  |  |  |  |
| TFT |  |  |  |  | TFT stable |  |  |  |  |
| TWTH |  |  |  | TFT stable |  |  |  |  |  |
| SDH |  |  |  |  |  |  |  |  |  |
| SDD |  |  |  |  |  |  |  | GDA stable |  |
| GDA |  |  |  |  |  |  | GDA stable |  |  |
| P |  |  |  |  |  |  |  |  |  |

Appendix table 29. Pairwise contest results for 9 strategies when b =1, c = 0.5, n = 100, e = 0.01.

|  | AC | AD | AT | TFT | TWTH | SDH | SDD | GDA | P |
| --- | --- | --- | --- | --- | --- | --- | --- | --- | --- |
| AC |  |  |  |  |  |  |  |  |  |
| AD |  |  |  |  |  |  |  |  |  |
| AT |  |  |  |  |  |  |  |  | P stable |
| TFT |  |  |  |  |  |  |  |  |  |
| TWTH |  |  |  |  |  |  |  |  |  |
| SDH |  |  |  |  |  |  |  |  |  |
| SDD |  |  |  |  |  |  |  |  |  |
| GDA |  |  |  |  |  |  |  |  |  |
| P |  |  | P stable |  |  |  |  |  |  |

Appendix table 30. Pairwise contest results for 9 strategies when b =1, c = 0.5, n = 100, e = 0.05.

|  | AC | AD | AT | TFT | TWTH | SDH | SDD | GDA | P |
| --- | --- | --- | --- | --- | --- | --- | --- | --- | --- |
| AC |  |  |  |  |  |  |  |  |  |
| AD |  |  |  |  |  |  |  |  |  |
| AT |  |  |  |  |  |  |  |  |  |
| TFT |  |  |  |  |  |  | SDD stable |  |  |
| TWTH |  |  |  |  |  |  |  |  |  |
| SDH |  |  |  |  |  |  |  |  |  |
| SDD |  |  |  | SDD stable |  |  |  |  |  |
| GDA |  |  |  |  |  |  |  |  |  |
| P |  |  |  |  |  |  |  |  |  |

Appendix table 31. Pairwise contest results for 9 strategies when b =1, c = 0.5, n = 100, e = 0.1.

|  | AC | AD | AT | TFT | TWTH | SDH | SDD | GDA | P |
| --- | --- | --- | --- | --- | --- | --- | --- | --- | --- |
| AC |  |  |  |  |  |  |  |  |  |
| AD |  |  |  |  |  |  |  |  |  |
| AT |  |  |  |  | AT stable |  |  |  |  |
| TFT |  |  |  |  |  |  | SDD stable | TFT stable |  |
| TWTH |  |  | AT stable |  |  |  | SDD stable |  | P stable |
| SDH |  |  |  |  |  |  |  |  |  |
| SDD |  |  |  | SDD stable | SDD stable |  |  |  |  |
| GDA |  |  |  | TFT stable |  |  |  |  |  |
| P |  |  |  |  | P stable |  |  |  |  |

Appendix table 32. Pairwise contest results for 9 strategies when b =1, c = 0.5, n = 100, e = 0.2.

|  | AC | AD | AT | TFT | TWTH | SDH | SDD | GDA | P |
| --- | --- | --- | --- | --- | --- | --- | --- | --- | --- |
| AC |  |  |  |  |  |  |  |  |  |
| AD |  |  |  |  |  | AD stable |  |  |  |
| AT |  |  |  |  |  |  |  | AT stable |  |
| TFT |  |  |  |  |  |  | SDD stable |  |  |
| TWTH |  |  |  |  |  |  |  |  |  |
| SDH |  | AD stable |  |  |  |  |  |  |  |
| SDD |  |  |  | SDD stable |  |  |  |  |  |
| GDA |  |  | AT stable |  |  |  |  |  | P stable |
| P |  |  |  |  |  |  |  | P stable |  |

Appendix table 33. Pairwise contest results for 9 strategies when b =1, c = 0.25, n = 10, e = 0.01.

|  | AC | AD | AT | TFT | TWTH | SDH | SDD | GDA | P |
| --- | --- | --- | --- | --- | --- | --- | --- | --- | --- |
| AC |  |  |  |  |  |  |  |  |  |
| AD |  |  |  |  |  |  |  |  |  |
| AT |  |  |  |  |  |  | SDD stable |  | P stable |
| TFT |  |  |  |  |  |  |  |  |  |
| TWTH |  |  |  |  |  |  |  |  |  |
| SDH |  |  |  |  |  |  |  |  |  |
| SDD |  |  | SDD stable |  |  |  |  |  |  |
| GDA |  |  |  |  |  |  |  |  |  |
| P |  |  | P stable |  |  |  |  |  |  |

Appendix table 34. Pairwise contest results for 9 strategies when b =1, c = 0.25, n = 10, e = 0.05.

|  | AC | AD | AT | TFT | TWTH | SDH | SDD | GDA | P |
| --- | --- | --- | --- | --- | --- | --- | --- | --- | --- |
| AC |  |  |  |  |  |  |  |  |  |
| AD |  |  |  |  |  |  |  |  |  |
| AT |  |  |  |  |  |  |  | GDA stable |  |
| TFT |  |  |  |  |  |  |  |  |  |
| TWTH |  |  |  |  |  |  |  |  |  |
| SDH |  |  |  |  |  |  |  |  |  |
| SDD |  |  |  |  |  |  |  |  |  |
| GDA |  |  | GDA stable |  |  |  |  |  |  |
| P |  |  |  |  |  |  |  |  |  |

Appendix table 35. Pairwise contest results for 9 strategies when b =1, c = 0.25, n = 10, e = 0.1.

|  | AC | AD | AT | TFT | TWTH | SDH | SDD | GDA | P |
| --- | --- | --- | --- | --- | --- | --- | --- | --- | --- |
| AC |  |  |  |  |  | SDH stable |  |  |  |
| AD |  |  |  |  |  |  |  |  |  |
| AT |  |  |  |  | TWTH stable |  |  |  | P stable |
| TFT |  |  |  |  |  |  |  |  |  |
| TWTH |  |  | TWTH stable |  |  |  |  |  |  |
| SDH | SDH stable |  |  |  |  |  | SDD stable |  |  |
| SDD |  |  |  |  |  | SDD stable |  |  |  |
| GDA |  |  |  |  |  |  |  |  |  |
| P |  |  | P stable |  |  |  |  |  |  |

Appendix table 36. Pairwise contest results for 9 strategies when b =1, c = 0.25, n = 10, e = 0.2.

|  | AC | AD | AT | TFT | TWTH | SDH | SDD | GDA | P |
| --- | --- | --- | --- | --- | --- | --- | --- | --- | --- |
| AC |  |  |  |  |  | SDH stable |  |  |  |
| AD |  |  |  |  |  |  |  |  |  |
| AT |  |  |  |  |  |  |  | GDA stable | P stable |
| TFT |  |  |  |  |  |  |  |  | TFT stable |
| TWTH |  |  |  |  |  |  |  |  |  |
| SDH | SDH stable |  |  |  |  |  |  |  |  |
| SDD |  |  |  |  |  |  |  |  |  |
| GDA |  |  | GDA stable |  |  |  |  |  |  |
| P |  |  | P stable | TFT stable |  |  |  |  |  |

Appendix table 37. Pairwise contest results for 9 strategies when b =1, c = 0.25, n = 20, e = 0.01.

|  | AC | AD | AT | TFT | TWTH | SDH | SDD | GDA | P |
| --- | --- | --- | --- | --- | --- | --- | --- | --- | --- |
| AC |  |  |  |  |  |  |  |  |  |
| AD |  |  |  |  |  |  |  |  |  |
| AT |  |  |  |  |  |  |  |  |  |
| TFT |  |  |  |  |  |  |  |  |  |
| TWTH |  |  |  |  |  |  |  |  |  |
| SDH |  |  |  |  |  |  |  |  |  |
| SDD |  |  |  |  |  |  |  |  |  |
| GDA |  |  |  |  |  |  |  |  |  |
| P |  |  |  |  |  |  |  |  |  |

Appendix table 38. Pairwise contest results for 9 strategies when b =1, c = 0.25, n = 20, e = 0.05.

|  | AC | AD | AT | TFT | TWTH | SDH | SDD | GDA | P |
| --- | --- | --- | --- | --- | --- | --- | --- | --- | --- |
| AC |  |  |  |  |  |  |  |  |  |
| AD |  |  |  |  |  |  |  |  |  |
| AT |  |  |  |  |  |  |  |  | P stable |
| TFT |  |  |  |  |  |  |  |  |  |
| TWTH |  |  |  |  |  |  |  |  |  |
| SDH |  |  |  |  |  |  |  |  |  |
| SDD |  |  |  |  |  |  |  |  |  |
| GDA |  |  |  |  |  |  |  |  |  |
| P |  |  | P stable |  |  |  |  |  |  |

Appendix table 39. Pairwise contest results for 9 strategies when b =1, c = 0.25, n = 20, e = 0.1.

|  | AC | AD | AT | TFT | TWTH | SDH | SDD | GDA | P |
| --- | --- | --- | --- | --- | --- | --- | --- | --- | --- |
| AC |  |  |  |  |  |  |  |  |  |
| AD |  |  |  |  |  |  |  |  |  |
| AT |  |  |  |  |  | SDH stable |  |  | P stable |
| TFT |  |  |  |  |  |  |  |  |  |
| TWTH |  |  |  |  |  |  |  |  |  |
| SDH |  |  | SDH stable |  |  |  |  |  |  |
| SDD |  |  |  |  |  |  |  |  |  |
| GDA |  |  |  |  |  |  |  |  |  |
| P |  |  | P stable |  |  |  |  |  |  |

Appendix table 40. Pairwise contest results for 9 strategies when b =1, c = 0.25, n = 20, e = 0.2.

|  | AC | AD | AT | TFT | TWTH | SDH | SDD | GDA | P |
| --- | --- | --- | --- | --- | --- | --- | --- | --- | --- |
| AC |  |  |  |  |  | SDH stable |  |  |  |
| AD |  |  |  |  |  |  |  |  |  |
| AT |  |  |  |  |  |  |  | GDA stable |  |
| TFT |  |  |  |  |  |  |  |  |  |
| TWTH |  |  |  |  |  |  |  |  |  |
| SDH | SDH stable |  |  |  |  |  |  |  |  |
| SDD |  |  |  |  |  |  |  |  |  |
| GDA |  |  | GDA stable |  |  |  |  |  |  |
| P |  |  |  |  |  |  |  |  |  |

Appendix table 41. Pairwise contest results for 9 strategies when b =1, c = 0.25, n = 50, e = 0.01.

|  | AC | AD | AT | TFT | TWTH | SDH | SDD | GDA | P |
| --- | --- | --- | --- | --- | --- | --- | --- | --- | --- |
| AC |  |  |  |  |  |  |  |  |  |
| AD |  |  |  |  |  |  |  |  |  |
| AT |  |  |  |  |  |  |  |  |  |
| TFT |  |  |  |  |  |  |  |  |  |
| TWTH |  |  |  |  |  |  |  |  |  |
| SDH |  |  |  |  |  |  |  |  |  |
| SDD |  |  |  |  |  |  |  |  |  |
| GDA |  |  |  |  |  |  |  |  |  |
| P |  |  |  |  |  |  |  |  |  |

Appendix table 42. Pairwise contest results for 9 strategies when b =1, c = 0.25, n = 50, e = 0.05.

|  | AC | AD | AT | TFT | TWTH | SDH | SDD | GDA | P |
| --- | --- | --- | --- | --- | --- | --- | --- | --- | --- |
| AC |  |  |  |  |  |  |  |  |  |
| AD |  |  |  |  |  |  |  |  |  |
| AT |  |  |  |  |  |  |  |  |  |
| TFT |  |  |  |  |  |  | TFT stable |  |  |
| TWTH |  |  |  |  |  |  |  |  |  |
| SDH |  |  |  |  |  |  |  |  |  |
| SDD |  |  |  | TFT stable |  |  |  |  |  |
| GDA |  |  |  |  |  |  |  |  |  |
| P |  |  |  |  |  |  |  |  |  |

Appendix table 43. Pairwise contest results for 9 strategies when b =1, c = 0.25, n = 50, e = 0.1.

|  | AC | AD | AT | TFT | TWTH | SDH | SDD | GDA | P |
| --- | --- | --- | --- | --- | --- | --- | --- | --- | --- |
| AC |  |  |  |  |  |  |  |  |  |
| AD |  |  |  |  |  |  |  |  |  |
| AT |  |  |  |  |  |  |  |  |  |
| TFT |  |  |  |  |  |  |  |  |  |
| TWTH |  |  |  |  |  |  |  |  |  |
| SDH |  |  |  |  |  |  |  |  |  |
| SDD |  |  |  |  |  |  |  |  |  |
| GDA |  |  |  |  |  |  |  |  |  |
| P |  |  |  |  |  |  |  |  |  |

Appendix table 44. Pairwise contest results for 9 strategies when b =1, c = 0.25, n = 50, e = 0.2.

|  | AC | AD | AT | TFT | TWTH | SDH | SDD | GDA | P |
| --- | --- | --- | --- | --- | --- | --- | --- | --- | --- |
| AC |  |  |  |  |  |  |  |  |  |
| AD |  |  |  |  |  |  |  |  |  |
| AT |  |  |  |  |  |  |  |  |  |
| TFT |  |  |  |  |  |  |  |  |  |
| TWTH |  |  |  |  |  |  |  |  |  |
| SDH |  |  |  |  |  |  |  |  |  |
| SDD |  |  |  |  |  |  |  |  |  |
| GDA |  |  |  |  |  |  |  |  | GDA stable |
| P |  |  |  |  |  |  |  | GDA stable |  |

Appendix table 45. Pairwise contest results for 9 strategies when b =1, c = 0.25, n = 100, e = 0.01.

|  | AC | AD | AT | TFT | TWTH | SDH | SDD | GDA | P |
| --- | --- | --- | --- | --- | --- | --- | --- | --- | --- |
| AC |  |  |  |  |  |  |  |  |  |
| AD |  |  |  |  |  |  |  |  |  |
| AT |  |  |  |  |  |  |  |  |  |
| TFT |  |  |  |  |  |  |  |  |  |
| TWTH |  |  |  |  |  |  |  |  |  |
| SDH |  |  |  |  |  |  |  |  |  |
| SDD |  |  |  |  |  |  |  |  |  |
| GDA |  |  |  |  |  |  |  |  |  |
| P |  |  |  |  |  |  |  |  |  |

Appendix table 46. Pairwise contest results for 9 strategies when b =1, c = 0.25, n = 100, e = 0.05.

|  | AC | AD | AT | TFT | TWTH | SDH | SDD | GDA | P |
| --- | --- | --- | --- | --- | --- | --- | --- | --- | --- |
| AC |  |  |  |  |  |  |  |  |  |
| AD |  |  |  |  |  |  |  |  |  |
| AT |  |  |  |  |  |  |  |  | P stable |
| TFT |  |  |  |  |  |  |  |  |  |
| TWTH |  |  |  |  |  |  |  |  |  |
| SDH |  |  |  |  |  |  |  |  |  |
| SDD |  |  |  |  |  |  |  |  |  |
| GDA |  |  |  |  |  |  |  |  |  |
| P |  |  | P stable |  |  |  |  |  |  |

Appendix table 47. Pairwise contest results for 9 strategies when b =1, c = 0.25, n = 100, e = 0.1.

|  | AC | AD | AT | TFT | TWTH | SDH | SDD | GDA | P |
| --- | --- | --- | --- | --- | --- | --- | --- | --- | --- |
| AC |  |  |  |  |  |  |  |  |  |
| AD |  |  |  |  |  |  |  |  |  |
| AT |  |  |  |  |  |  |  |  |  |
| TFT |  |  |  |  |  |  |  |  |  |
| TWTH |  |  |  |  |  |  |  |  |  |
| SDH |  |  |  |  |  |  |  |  |  |
| SDD |  |  |  |  |  |  |  |  |  |
| GDA |  |  |  |  |  |  |  |  |  |
| P |  |  |  |  |  |  |  |  |  |

Appendix table 48. Pairwise contest results for 9 strategies when b =1, c = 0.25, n = 100, e = 0.2.

|  | AC | AD | AT | TFT | TWTH | SDH | SDD | GDA | P |
| --- | --- | --- | --- | --- | --- | --- | --- | --- | --- |
| AC |  |  |  |  |  |  |  |  |  |
| AD |  |  |  |  |  |  |  |  |  |
| AT |  |  |  |  |  |  |  |  |  |
| TFT |  |  |  |  |  |  |  |  | TFT stable |
| TWTH |  |  |  |  |  |  |  |  | TWTH stable |
| SDH |  |  |  |  |  |  |  |  |  |
| SDD |  |  |  |  |  |  |  |  |  |
| GDA |  |  |  |  |  |  |  |  |  |
| P |  |  |  | TFT stable | TWTH stable |  |  |  |  |

Appendix table 49. Pairwise contest results for generous TFT, when b =1, c = 0.75, n = 10, e = 0.01.

|  | AC | AD | AT | TFT | TWTH | SDH | SDD | GDA | P |
| --- | --- | --- | --- | --- | --- | --- | --- | --- | --- |
| GTFT |  |  |  |  |  |  |  |  |  |

Appendix table 50. Pairwise contest results for generous TFT, when b =1, c = 0.75, n = 10, e = 0.05.

|  | AC | AD | AT | TFT | TWTH | SDH | SDD | GDA | P |
| --- | --- | --- | --- | --- | --- | --- | --- | --- | --- |
| GTFT |  |  |  |  |  | SDH  stable |  |  |  |

Appendix table 51. Pairwise contest results for generous TFT, when b =1, c = 0.75, n = 10, e = 0. 1.

|  | AC | AD | AT | TFT | TWTH | SDH | SDD | GDA | P |
| --- | --- | --- | --- | --- | --- | --- | --- | --- | --- |
| GTFT |  |  |  |  |  | SDH  stable |  |  |  |

Appendix table 52. Pairwise contest results for generous TFT, when b =1, c = 0.75, n = 10, e = 0.2.

|  | AC | AD | AT | TFT | TWTH | SDH | SDD | GDA | P |
| --- | --- | --- | --- | --- | --- | --- | --- | --- | --- |
| GTFT |  |  |  |  |  | SDH  stable |  |  |  |

Appendix table 53. Pairwise contest results for generous TFT, when b =1, c = 0.75, n = 20, e = 0.01.

|  | AC | AD | AT | TFT | TWTH | SDH | SDD | GDA | P |
| --- | --- | --- | --- | --- | --- | --- | --- | --- | --- |
| GTFT |  |  |  |  |  |  |  |  |  |

Appendix table 54. Pairwise contest results for generous TFT, when b =1, c = 0.75, n = 20, e = 0.05.

|  | AC | AD | AT | TFT | TWTH | SDH | SDD | GDA | P |
| --- | --- | --- | --- | --- | --- | --- | --- | --- | --- |
| GTFT |  |  |  |  |  | SDH  stable |  |  |  |

Appendix table 55. Pairwise contest results for generous TFT, when b =1, c = 0.75, n = 20, e = 0. 1.

|  | AC | AD | AT | TFT | TWTH | SDH | SDD | GDA | P |
| --- | --- | --- | --- | --- | --- | --- | --- | --- | --- |
| GTFT |  |  |  |  |  | SDH  stable |  |  |  |

Appendix table 56. Pairwise contest results for generous TFT, when b =1, c = 0.75, n = 20, e = 0.2.

|  | AC | AD | AT | TFT | TWTH | SDH | SDD | GDA | P |
| --- | --- | --- | --- | --- | --- | --- | --- | --- | --- |
| GTFT |  |  |  |  |  | SDH  stable |  |  |  |

Appendix table 57. Pairwise contest results for generous TFT, when b =1, c = 0.75, n = 50, e = 0.01.

|  | AC | AD | AT | TFT | TWTH | SDH | SDD | GDA | P |
| --- | --- | --- | --- | --- | --- | --- | --- | --- | --- |
| GTFT |  |  |  |  |  |  |  |  |  |

Appendix table 58. Pairwise contest results for generous TFT, when b =1, c = 0.75, n = 50, e = 0.05.

|  | AC | AD | AT | TFT | TWTH | SDH | SDD | GDA | P |
| --- | --- | --- | --- | --- | --- | --- | --- | --- | --- |
| GTFT |  |  |  |  |  |  |  |  |  |

Appendix table 59. Pairwise contest results for generous TFT, when b =1, c = 0.75, n = 50, e = 0. 1.

|  | AC | AD | AT | TFT | TWTH | SDH | SDD | GDA | P |
| --- | --- | --- | --- | --- | --- | --- | --- | --- | --- |
| GTFT |  |  |  |  |  |  |  |  |  |

Appendix table 60. Pairwise contest results for generous TFT, when b =1, c = 0.75, n = 50, e = 0.2.

|  | AC | AD | AT | TFT | TWTH | SDH | SDD | GDA | P |
| --- | --- | --- | --- | --- | --- | --- | --- | --- | --- |
| GTFT |  |  |  |  |  |  |  |  |  |

Appendix table 61. Pairwise contest results for generous TFT, when b =1, c = 0.75, n = 100, e = 0.01.

|  | AC | AD | AT | TFT | TWTH | SDH | SDD | GDA | P |
| --- | --- | --- | --- | --- | --- | --- | --- | --- | --- |
| GTFT |  |  |  |  |  |  |  |  |  |

Appendix table 62. Pairwise contest results for generous TFT, when b =1, c = 0.75, n = 100, e = 0.05.

|  | AC | AD | AT | TFT | TWTH | SDH | SDD | GDA | P |
| --- | --- | --- | --- | --- | --- | --- | --- | --- | --- |
| GTFT |  |  |  |  |  |  |  |  |  |

Appendix table 63. Pairwise contest results for generous TFT, when b =1, c = 0.75, n = 100, e = 0. 1.

|  | AC | AD | AT | TFT | TWTH | SDH | SDD | GDA | P |
| --- | --- | --- | --- | --- | --- | --- | --- | --- | --- |
| GTFT |  |  |  |  |  |  |  |  |  |

Appendix table 64. Pairwise contest results for generous TFT, when b =1, c = 0.75, n = 100, e = 0.2.

|  | AC | AD | AT | TFT | TWTH | SDH | SDD | GDA | P |
| --- | --- | --- | --- | --- | --- | --- | --- | --- | --- |
| GTFT |  |  |  |  |  |  |  |  |  |

Appendix table 65. Pairwise contest results for generous TFT, when b =1, c = 0.5, n = 10, e = 0.01.

|  | AC | AD | AT | TFT | TWTH | SDH | SDD | GDA | P |
| --- | --- | --- | --- | --- | --- | --- | --- | --- | --- |
| GTFT |  |  |  |  |  |  |  |  |  |

Appendix table 66. Pairwise contest results for generous TFT, when b =1, c = 0.5, n = 10, e = 0.05.

|  | AC | AD | AT | TFT | TWTH | SDH | SDD | GDA | P |
| --- | --- | --- | --- | --- | --- | --- | --- | --- | --- |
| GTFT |  |  |  |  |  | SDH  stable |  |  |  |

Appendix table 67. Pairwise contest results for generous TFT, when b =1, c = 0.5, n = 10, e = 0. 1.

|  | AC | AD | AT | TFT | TWTH | SDH | SDD | GDA | P |
| --- | --- | --- | --- | --- | --- | --- | --- | --- | --- |
| GTFT |  |  |  |  |  | SDH  stable |  |  |  |

Appendix table 68. Pairwise contest results for generous TFT, when b =1, c = 0.5, n = 10, e = 0.2.

|  | AC | AD | AT | TFT | TWTH | SDH | SDD | GDA | P |
| --- | --- | --- | --- | --- | --- | --- | --- | --- | --- |
| GTFT |  |  |  |  |  | SDH  stable |  |  |  |

Appendix table 69. Pairwise contest results for generous TFT, when b =1, c = 0.5, n = 20, e = 0.01.

|  | AC | AD | AT | TFT | TWTH | SDH | SDD | GDA | P |
| --- | --- | --- | --- | --- | --- | --- | --- | --- | --- |
| GTFT |  |  |  |  |  |  |  |  |  |

Appendix table 70. Pairwise contest results for generous TFT, when b =1, c = 0.5, n = 20, e = 0.05.

|  | AC | AD | AT | TFT | TWTH | SDH | SDD | GDA | P |
| --- | --- | --- | --- | --- | --- | --- | --- | --- | --- |
| GTFT |  |  |  |  |  |  |  |  |  |

Appendix table 71. Pairwise contest results for generous TFT, when b =1, c = 0.5, n = 20, e = 0. 1.

|  | AC | AD | AT | TFT | TWTH | SDH | SDD | GDA | P |
| --- | --- | --- | --- | --- | --- | --- | --- | --- | --- |
| GTFT |  |  |  |  |  |  |  |  |  |

Appendix table 72. Pairwise contest results for generous TFT, when b =1, c = 0.5, n = 20, e = 0.2.

|  | AC | AD | AT | TFT | TWTH | SDH | SDD | GDA | P |
| --- | --- | --- | --- | --- | --- | --- | --- | --- | --- |
| GTFT |  |  |  |  |  | SDH  stable |  |  |  |

Appendix table 73. Pairwise contest results for generous TFT, when b =1, c = 0.5, n = 50, e = 0.01.

|  | AC | AD | AT | TFT | TWTH | SDH | SDD | GDA | P |
| --- | --- | --- | --- | --- | --- | --- | --- | --- | --- |
| GTFT |  |  |  |  |  |  |  |  |  |

Appendix table 74. Pairwise contest results for generous TFT, when b =1, c = 0.5, n = 50, e = 0.05.

|  | AC | AD | AT | TFT | TWTH | SDH | SDD | GDA | P |
| --- | --- | --- | --- | --- | --- | --- | --- | --- | --- |
| GTFT |  |  |  |  |  |  |  |  |  |

Appendix table 75. Pairwise contest results for generous TFT, when b =1, c = 0.5, n = 50, e = 0. 1.

|  | AC | AD | AT | TFT | TWTH | SDH | SDD | GDA | P |
| --- | --- | --- | --- | --- | --- | --- | --- | --- | --- |
| GTFT |  |  |  |  |  |  |  |  |  |

Appendix table 76. Pairwise contest results for generous TFT, when b =1, c = 0.5, n = 50, e = 0.2.

|  | AC | AD | AT | TFT | TWTH | SDH | SDD | GDA | P |
| --- | --- | --- | --- | --- | --- | --- | --- | --- | --- |
| GTFT |  |  |  |  |  |  |  |  |  |

Appendix table 77. Pairwise contest results for generous TFT, when b =1, c = 0.5, n = 100, e = 0.01.

|  | AC | AD | AT | TFT | TWTH | SDH | SDD | GDA | P |
| --- | --- | --- | --- | --- | --- | --- | --- | --- | --- |
| GTFT |  |  |  |  |  |  |  |  |  |

Appendix table 78. Pairwise contest results for generous TFT, when b =1, c = 0.5, n = 100, e = 0.05.

|  | AC | AD | AT | TFT | TWTH | SDH | SDD | GDA | P |
| --- | --- | --- | --- | --- | --- | --- | --- | --- | --- |
| GTFT |  |  |  |  |  |  |  |  |  |

Appendix table 79. Pairwise contest results for generous TFT, when b =1, c = 0.5, n = 100, e = 0. 1.

|  | AC | AD | AT | TFT | TWTH | SDH | SDD | GDA | P |
| --- | --- | --- | --- | --- | --- | --- | --- | --- | --- |
| GTFT |  |  |  |  |  |  |  |  |  |

Appendix table 80. Pairwise contest results for generous TFT, when b =1, c = 0.5, n = 100, e = 0.2.

|  | AC | AD | AT | TFT | TWTH | SDH | SDD | GDA | P |
| --- | --- | --- | --- | --- | --- | --- | --- | --- | --- |
| GTFT |  |  |  |  |  |  |  |  |  |

Appendix table 81. Pairwise contest results for generous TFT, when b =1, c = 0.25, n = 10, e = 0.01.

|  | AC | AD | AT | TFT | TWTH | SDH | SDD | GDA | P |
| --- | --- | --- | --- | --- | --- | --- | --- | --- | --- |
| GTFT |  |  |  |  |  |  |  |  |  |

Appendix table 82. Pairwise contest results for generous TFT, when b =1, c = 0.25, n = 10, e = 0.05.

|  | AC | AD | AT | TFT | TWTH | SDH | SDD | GDA | P |
| --- | --- | --- | --- | --- | --- | --- | --- | --- | --- |
| GTFT |  |  |  |  |  |  |  |  |  |

Appendix table 83. Pairwise contest results for generous TFT, when b =1, c = 0.25, n = 10, e = 0. 1.

|  | AC | AD | AT | TFT | TWTH | SDH | SDD | GDA | P |
| --- | --- | --- | --- | --- | --- | --- | --- | --- | --- |
| GTFT |  |  |  |  |  | SDH  stable |  |  |  |

Appendix table 84. Pairwise contest results for generous TFT, when b =1, c = 0.25, n = 10, e = 0.2.

|  | AC | AD | AT | TFT | TWTH | SDH | SDD | GDA | P |
| --- | --- | --- | --- | --- | --- | --- | --- | --- | --- |
| GTFT |  |  |  |  |  | SDH  stable |  |  |  |

Appendix table 85. Pairwise contest results for generous TFT, when b =1, c = 0.25, n = 20, e = 0.01.

|  | AC | AD | AT | TFT | TWTH | SDH | SDD | GDA | P |
| --- | --- | --- | --- | --- | --- | --- | --- | --- | --- |
| GTFT |  |  |  |  |  |  |  |  |  |

Appendix table 86. Pairwise contest results for generous TFT, when b =1, c = 0.25, n = 20, e = 0.05.

|  | AC | AD | AT | TFT | TWTH | SDH | SDD | GDA | P |
| --- | --- | --- | --- | --- | --- | --- | --- | --- | --- |
| GTFT |  |  |  |  |  |  |  |  |  |

Appendix table 87. Pairwise contest results for generous TFT, when b =1, c = 0.25, n = 20, e = 0. 1.

|  | AC | AD | AT | TFT | TWTH | SDH | SDD | GDA | P |
| --- | --- | --- | --- | --- | --- | --- | --- | --- | --- |
| GTFT |  |  |  |  |  |  |  |  |  |

Appendix table 88. Pairwise contest results for generous TFT, when b =1, c = 0.25, n = 20, e = 0.2.

|  | AC | AD | AT | TFT | TWTH | SDH | SDD | GDA | P |
| --- | --- | --- | --- | --- | --- | --- | --- | --- | --- |
| GTFT |  |  |  |  |  | SDH  stable |  |  |  |

Appendix table 89. Pairwise contest results for generous TFT, when b =1, c = 0.25, n = 50, e = 0.01.

|  | AC | AD | AT | TFT | TWTH | SDH | SDD | GDA | P |
| --- | --- | --- | --- | --- | --- | --- | --- | --- | --- |
| GTFT |  |  |  |  |  |  |  |  |  |

Appendix table 90. Pairwise contest results for generous TFT, when b =1, c = 0.25, n = 50, e = 0.05.

|  | AC | AD | AT | TFT | TWTH | SDH | SDD | GDA | P |
| --- | --- | --- | --- | --- | --- | --- | --- | --- | --- |
| GTFT |  |  |  |  |  |  |  |  |  |

Appendix table 91. Pairwise contest results for generous TFT, when b =1, c = 0.25, n = 50, e = 0. 1.

|  | AC | AD | AT | TFT | TWTH | SDH | SDD | GDA | P |
| --- | --- | --- | --- | --- | --- | --- | --- | --- | --- |
| GTFT |  |  |  |  |  |  |  |  |  |

Appendix table 92. Pairwise contest results for generous TFT, when b =1, c = 0.25, n = 50, e = 0.2.

|  | AC | AD | AT | TFT | TWTH | SDH | SDD | GDA | P |
| --- | --- | --- | --- | --- | --- | --- | --- | --- | --- |
| GTFT |  |  |  |  |  |  |  |  |  |

Appendix table 93. Pairwise contest results for generous TFT, when b =1, c = 0.25, n = 100, e = 0.01.

|  | AC | AD | AT | TFT | TWTH | SDH | SDD | GDA | P |
| --- | --- | --- | --- | --- | --- | --- | --- | --- | --- |
| GTFT |  |  |  |  |  |  |  |  |  |

Appendix table 94. Pairwise contest results for generous TFT, when b =1, c = 0.25, n = 100, e = 0.05.

|  | AC | AD | AT | TFT | TWTH | SDH | SDD | GDA | P |
| --- | --- | --- | --- | --- | --- | --- | --- | --- | --- |
| GTFT |  |  |  |  |  |  |  |  |  |

Appendix table 95. Pairwise contest results for generous TFT, when b =1, c = 0.25, n = 100, e = 0. 1.

|  | AC | AD | AT | TFT | TWTH | SDH | SDD | GDA | P |
| --- | --- | --- | --- | --- | --- | --- | --- | --- | --- |
| GTFT |  |  |  |  |  |  |  |  |  |

Appendix table 96. Pairwise contest results for generous TFT, when b =1, c = 0.25, n = 100, e = 0.2.

|  | AC | AD | AT | TFT | TWTH | SDH | SDD | GDA | P |
| --- | --- | --- | --- | --- | --- | --- | --- | --- | --- |
| GTFT |  |  |  |  |  |  |  |  |  |
